# Constructing stochastic models from deterministic process equations by propensity adjustment

**DOI:** 10.1186/1752-0509-5-187

**Published:** 2011-11-08

**Authors:** Jialiang Wu, Brani Vidakovic, Eberhard O Voit

**Affiliations:** 1 Deparment of Mathematics, Bioinformatics Program, Georgia Institute of Technology, Atlanta, GA30332, USA; 2 The Wallace H. Coulter Department of Biomedical Engineering, Georgia Institute of Technology, Atlanta, GA30332, USA; 3 Integrative BioSystems Institute and The Wallace H. Coulter Department of Biomedical Engineering, Georgia Institute of Technology, Atlanta, GA30332, USA

## Abstract

**Background:**

Gillespie's stochastic simulation algorithm (SSA) for chemical reactions admits three kinds of elementary processes, namely, mass action reactions of 0^th^, 1^st ^or 2^nd ^order. All other types of reaction processes, for instance those containing non-integer kinetic orders or following other types of kinetic laws, are assumed to be convertible to one of the three elementary kinds, so that SSA can validly be applied. However, the conversion to elementary reactions is often difficult, if not impossible. Within deterministic contexts, a strategy of model reduction is often used. Such a reduction simplifies the actual system of reactions by merging or approximating intermediate steps and omitting reactants such as transient complexes. It would be valuable to adopt a similar reduction strategy to stochastic modelling. Indeed, efforts have been devoted to manipulating the chemical master equation (CME) in order to achieve a proper propensity function for a reduced stochastic system. However, manipulations of CME are almost always complicated, and successes have been limited to relative simple cases.

**Results:**

We propose a rather general strategy for converting a deterministic process model into a corresponding stochastic model and characterize the mathematical connections between the two. The deterministic framework is assumed to be a generalized mass action system and the stochastic analogue is in the format of the chemical master equation. The analysis identifies situations: where a direct conversion is valid; where internal noise affecting the system needs to be taken into account; and where the propensity function must be mathematically adjusted. The conversion from deterministic to stochastic models is illustrated with several representative examples, including reversible reactions with feedback controls, Michaelis-Menten enzyme kinetics, a genetic regulatory motif, and stochastic focusing.

**Conclusions:**

The construction of a stochastic model for a biochemical network requires the utilization of information associated with an equation-based model. The conversion strategy proposed here guides a model design process that ensures a valid transition between deterministic and stochastic models.

## Background

Most stochastic models of biochemical reactions are based on the fundamental assumption that no more than one reaction can occur at the exact same time. A consequence of this assumption is that only elementary chemical reactions can be converted directly into stochastic analogues [1]. These include: 1) zero-order reactions, such as the generation of molecules at a constant rate; 2) first-order reactions, with examples including elemental chemical reactions as well as transport and decay processes; and 3) second-order reactions, which include heterogeneous and homogeneous bimolecular reactions (dimerization). Reactions with integer kinetic orders other than 0, 1 and 2 are to be treated as combinations of sequential elementary reactions. The advantage of the premise of non-simultaneous reaction steps is that the stochastic reaction rate can be calculated from a deterministic, equation-based model with some degree of rigor, even though the derivation is usually not based on first physical principles but instead depends on other assumptions and on macroscopic information, such as a fixed rate constant in the equation-based model. The severe disadvantage is that this rigorous treatment is not practical for modelling larger biochemical reaction systems. The reasons include the following. First, in many cases, elementary reaction rates are not available. Secondly, even in the case that all reaction parameters are available, the computational expense is very significant when the system involves many species and reactions, and this fact ultimately leads to a combinatorial explosion of required computations. Within a deterministic modelling framework, the common practice in this situation is to fit the transient and steady-state experimental data with a phenomenological, (differential) equation-based model, which explicitly or implicitly eliminates or merges some intermediate species and reactions. The best-known examples are probably Michaelis-Menten and Hill rate laws, which are ultimately explicit, but in truth approximate a multivariate system of underlying chemical processes.

Similar model reduction efforts have been carried out for stochastic modelling. For instance, the use of a complex-order function (which corresponds to a reduced equation-based model) was shown to be justified for some types of stochastic simulations. A prominent example is again the Michaelis-Menten rate law, which can be reduced from a system of elementary reactions to an explicit function by means of the *quasi-steady-state assumption *(see Result section and [2,3]). However, model reduction within the stochastic framework has proven to be far more difficult than in the deterministic counterpart. The difficulties are mainly due to the fact that the reduction must be carried out on the chemical master equation (CME). This process is nontrivial and has succeeded only in simple cases.

In general, the construction of a stochastic model for a large biochemical network requires the use of information available from an equation-based model. In the past, several strategies have been proposed for this purpose and within the context of Gillespie's exact stochastic simulation algorithm (SSA; [1]) and its variants [4]. For example, Tian and Burrage [5] proposed that a stochastic model could be directly formulated from the deterministic model through a Poisson leaping procedure. However, a rigorous mathematical justification for such a conversion is lacking. Typical moment-based approaches [6-8] derive ODEs for the statistical moments of the stochastic model from an equation-based model where the 0^th^, 1^st ^and 2^nd ^order reactions follow mass action rate laws. More recently the moment method was extended to cover models consisting of rational rate laws [9]. Moreover, it was realized that the moment method is complementary to, but cannot fully replace, stochastic simulations, because it does not cover situations like genetic switches [6,10].

In this article, we explore the mathematical connection between deterministic and stochastic frameworks for the pertinent case of Generalized Mass Action (GMA) systems, which are frequently used in Biochemical Systems Theory (BST; [11-13]). Specifically, we address two questions: First, under what conditions can a deterministic, equation-based model be converted directly into a stochastic simulation model? And second, what is a proper way of implementing this conversion? We will develop a method to answer these questions and demonstrate it for functions in the canonical power-law format of GMA systems. However, the results are applicable to other functions and formats as well, as we will demonstrate with several examples.

### Representations of systems of biochemical reactions

Consider a well-stirred biochemical reaction system with constant volume and temperature, where *N_s _*different chemical species ${\left\{S_{s}\right\}}_{s=1}^{N_{s}}$, interact through *N_r _*unidirectional reaction channels ${\left\{R_{r}\right\}}_{r=1}^{N_{r}}$. Each reaction channel can be characterized as

$$R_{r}:{\underset{-}{v}}_{r1}S_{1}+\ldots +{\underset{-}{v}}_{rN_{s}}S_{N_{s}}\overset{k_{r}}{\underset{}{\to }}{\bar{v}}_{r1}S_{1}+\ldots +{\bar{v}}_{rN_{s}}S_{N_{s}},$$

where ${\underset{-}{v}}_{rs}$ and ${\bar{v}}_{rs}$ are the counts of molecular species *S_s _*consumed and produced due to reaction *R_r_*, respectively, and *k_r _*is the rate constant. The changed amount of $S_{s}v_{rs}={\bar{v}}_{rs}-{\underset{-}{v}}_{rs}$, which is due to the firing of reaction *R_r_*, defines the stoichiometric coefficient of *S_s _*in *R_r_*. The stoichiometric coefficients of all species can be summarized according to each reaction *R_r _*in the stoichiometric vector

$$v_{r}≜\left\{\begin{array}{c} v_{r1}\\ \vdots \\ v_{rN_{s}} \end{array}\right\}\in {\mathbb{Z}}^{N_{s}}.$$

The stoichiometric vectors of all reactions can further be arranged as the stoichiometric matrix of the system

$$V≜\left[v_{1},\ldots ,v_{N_{r}}\right]\in {\mathbb{Z}}^{N_{s}\times N_{r}}.$$

The size of the system is defined as Φ = *AU*, where *A *is the Avogadro number and *U *is the reaction volume.

The modelling of biochemical reaction networks typically uses one of two conceptual frameworks: deterministic or stochastic. In a deterministic framework, the state of the system is given by the a non-negative vector $\left[X\left(t\right)\right]={\left[\left[X_{1}\left(t\right)\right],\ldots ,\left[X_{N_{s}}\left(t\right)\right]\right]}^{T}\in {\mathbb{R}}^{N_{s}}$, where component [*X_s_*(*t*)] represents the concentration of species *S_s_*, measured in moles per unit volume. The temporal evolution of the state of the system is modelled by a set of ordinary differential equations, which in our case are assumed to follow a generalized mass action (GMA) kinetic law. By contrast, in a stochastic framework, the state of the systems is characterized by a vector $x\left(t\right)={\left[x_{1}\left(t\right),\ldots ,x_{N_{s}}\left(t\right)\right]}^{T}\in {\mathbb{Z}}^{N_{s}}$, whose values are non-negative integers. Specifically, *x_s_*(*t*) = Φ [*X_s _*(*t*)] is the count of *S_s _*molecules, which is a sample value of the random variable *X_s_*(*t*). The system dynamics of this process is typically described with the chemical master equation (CME). Both GMA and CME will be discussed in detail in the following sections.

### Motivation for the power-law formalism: reactions in crowded media

Power-law functions with non-integer kinetics have proven very useful in biochemical systems analysis, and forty years of research have demonstrated their wide applicability (*e.g*., see [11-13]). Generically, this type of description of a biochemical reaction can be seen either as a Taylor approximation in logarithmic space or as a heuristic or phenomenological model that has been applied successfully hundreds of times and in different contexts, even though it is difficult or impossible in many situations to trace it back to first mechanistic principles. A particularly interesting line of support for the power-law format can be seen in the example of a bimolecular reaction occurring in a spatially restricted environment. Savageau demonstrated that the kinetics of such a reaction can be validly formulated as a generalization of the law of mass action, where non-integer kinetic orders are allowed [14,15]. Neff and colleagues [16-18] showed with careful experiments that this formulation is actually more accurate than alternative approaches.

Within the conceptual framework of power-law representations, the rate of the association reaction between molecules of species *S*_1 _and *S*_2 _is given as $k{\left[X_{1}\left(t\right)\right]}^{f_{1}}{\left[X_{2}\left(t\right)\right]}^{f_{2}}$. Here, *k *is the *rate constant *and *f*_1 _and *f*_2 _are real-valued *kinetic orders*, which are no longer necessarily positive integers as it is assumed in a mass action law. As an example, consider the reversible bimolecular reaction $S_{1}+S_{2}\overset{k_{f}}{\underset{k_{b}}{⇌}}S_{3}$. Like Neff and colleagues [17], we begin by formulating a discrete update function for the population of *S*_3 _molecules as

(1) $$x_{3}\left(t+\Delta t\right)\;\text{ - }\;x_{3}\left(t\right)=f\left(\left[X_{1}\right],\left[X_{2}\right]\right)\Delta t\;x_{1}x_{2}\text{ - }g\left(\left[X_{3}\right]\right)\Delta tx_{3}.$$

The first term on the right-hand side of this equation, *f *([*X*_1_], [*X*_2_])Δ*t x*_1_*x*_2_, describes the production of *S*_3_: it depends on the totality of possible collisions *x*_1 _*x*_2 _and also on some fraction *f *([*X*_1_], [*X*_2_])Δ*t *that actually reacts and forms the product. In a dilute environment, *f *([*X*_1_], [*X*_2_]) equals a traditional rate constant, and the reaction obeys the law of mass action, while in a spatially restricted environment, such as the cytoplasm, one needs to take crowding effects into account. As shown in Savageau [14,15], the desired fraction of a reaction in a crowded environment becomes a *rate function *that depends on the current concentrations of *S*_1 _and *S*_2_. The second term, *g *([*X*_3_]) Δ*tx*_3_, describes the fraction *g *([*X*_3_]) Δ*t *of species *S*_3 _that dissociates back into *S*_1 _and *S*_2_. This fraction may depend on some functional form of [*X*_3_] because in a crowded environment the complex may not be able to dissociate effectively. Thus, rate constants in the generalized mass action setting become rate functions (*cf*. [17]).

By taking the limit Δt → 0, one obtains the differential equation

(2) $$\frac{dx_{3}}{dt}=f\left(\left[X_{1}\right],\left[X_{2}\right]\right)x_{1}x_{2}\text{ - }g\left(\left[X_{3}\right]\right)x_{3}.$$

Savageau used Taylor series expansion to approximate the functions *f *and *g *in the logarithmic space (log [*X*_1_], log[*X*_2_]) around some operating point (*a*, *b*). The result for *f *is

(3) $$\begin{array}{c} \log f\left(\left[X_{1}\right],\left[X_{2}\right]\right)≜F\left(\log\left[X_{1}\right],\log\left[X_{2}\right]\right)\\ ={\left(F\left(a,b\right)+\frac{\partial f\left(\left[X_{1}\right],\left[X_{2}\right]\right)}{\partial \left[X_{1}\right]}\right|}_{\left(a,b\right)}\left(\log\left[X_{1}\right]-a\right)\\ +{\left(\frac{\partial f\left(\left[X_{1}\right],\left[X_{2}\right]\right)}{\partial \left[X_{2}\right]}\right|}_{\left(a,b\right)}\left(\log\left[X_{2}\right]-b\right)+\text{HOT}\\ \approx k_{f}+\alpha \log\left[X_{1}\right]+\beta \log\left[X_{2}\right], \end{array}$$

where *k_f_*, *α*, and *β *are constants related to the chosen operating point (*a*, *b*). The final step is achieved by ignoring all higher order terms (HOT) beyond the constant and linear terms. Transformation back to the Cartesian space yields

(4) $$f\left(\left[X_{1}\right],\left[X_{2}\right]\right)\approx k_{a}{\left[X_{1}\right]}^{\alpha }{\left[X_{2}\right]}^{\beta },k_{a}=e^{k_{f}}.$$

The same procedure leads to the power-law expression for the degradation term: *g *([*X*_3_]) ≈ *k_d _*[*X*_3_]*^γ^*. By combining constants we arrive at a power-law representation for the dynamics of species *S*_3 _as

(5) $$\begin{array}{ccc} \frac{d\left[X_{3}\right]}{dt} & =k_{a}{\left[X_{1}\right]}^{\alpha }{\left[X_{2}\right]}^{\beta }\left[X_{1}\right]\left[X_{2}\right]-k_{d}{\left[X_{3}\right]}^{\gamma }\left[X_{3}\right] & \\ & =k_{a}{\left[X_{1}\right]}^{a}{\left[X_{2}\right]}^{b}-k_{d}{\left[X_{3}\right]}^{c}, \end{array}$$

where *a *= *α *+ 1, *b *= *β *+ 1, and *c *= *γ *+ 1. As long as *k_f_*, *k_d_*, *a*, *b *and *c *remain more or less constant throughout a relevant range, the power-law model is mathematically well justified. In actual applications, the values of rate constants and kinetic orders can be estimated from experimental data [19]. When the functions *f *and *g *are originally not in power-law format, they can be locally approximated by power-law functions with a procedure similar to the one shown above (Equations (3) to (5)). An illustration will be given in the example section.

### The Generalized Mass Action (GMA) format

In the GMA format within Biochemical Systems Theory, each process is represented as a univariate or multivariate power-law function. GMA models may be developed *de novo *or as an approximation of some other nonlinear rate laws. GMA models characterize the time evolution of the system state given that the system was in the state *X *(*t*_0_) at some initial time *t*_0_. Generically, the state of the system is changed within a sufficiently small time interval by one out of the *N_r _*possible reactions that can occur in the system. The reaction velocity through reaction channel *R_r _*is:

(6) $$\left[\frac{{\left[X_{1}\left(t\right)\right]}^{\prime }}{v_{r1}}=\ldots =\frac{{\left[X_{N_{s}}\left(t\right)\right]}^{\prime }}{v_{rN_{s}}}\right]=k_{r}\overset{N_{s}}{\underset{s=1}{\prod }}{\left[X_{s}\left(t\right)\right]}^{f_{rs}}$$

for those $v_{rs}={\bar{v}}_{rs}-{\underset{-}{v}}_{rs}\neq 0$, *s *= 1, ..., *N_s_*. As shown in the example of a bimolecular reaction, the kinetic order *f_rs _*associated with species *S_s _*captures the effects of both reactant properties (such as the stoichiometric coefficient *v_rs_*) and environmental influences (such as temperature, pressure, molecular crowding effects, etc.). Therefore *f_rs _*does not necessary equal an integer *v_rs_*, which is assumed to be the case in mass action kinetics, but is possibly real-valued and may be negative. Summing up the contributions of all reactions, one obtains a GMA model describing the dynamics of *S_s _*as

(7) $$\frac{d}{dt}\left[X_{s}\left(t\right)\right]=\overset{N_{r}}{\underset{r=1}{\sum }}v_{rs}k_{r}\overset{N_{s}}{\underset{s=1}{\prod }}{\left[X_{s}\left(t\right)\right]}^{f_{rs}}$$

for every *s *= 1, ..., *N_s_*. Each reaction contributes either a production flux or a degradation flux to the dynamics of a certain species. Positive terms (*v_rs _*> 0) represent the production of *S_s_*, while negative terms (*v_rs _*< 0) describe degradation. If *f_rs _*is positive, then *S_s _*accelerates the reaction *R_r_*; a negative value represents that *S_s _*inhibits the reaction, and *f_rs _*= 0 implies that *S_s _*has no influence on the reaction. The rate constant *k_r _*for reaction *R_r_*, is either positive or zero. Both, the rate constant and the kinetic order, are to be estimated from data.

### Proper use of equation-based functions for stochastic simulations

The fundamental concept of a stochastic simulation is the propensity function *α*(**X**), and *α*(**X**)*dt *describes the probability that a reaction will change the value of a system variable within the next (infinitesimal) time interval (*t*, *t *+*dt*). While a formal definition will be given later (Equation 18), it is easy to intuit that the propensity function is in some sense analogous to the rate in the corresponding deterministic model. In fact, the propensity function is traditionally assumed to be *α*(**X**) = *f_s_*(**X**), if the deterministic model is *X_s_*' = *f_s_*(**X**, *t*), *s *= 1, ..., *N_s_*. However, a proper justification for this common practice is by and large missing. Indeed, we will show that the direct use of a rate function as the propensity function in a stochastic simulation algorithm requires that at least one of the following assumptions be true:

1) *f *is a linear function;

2) the reaction is monomolecular;

3) all *X_i _*in the system are noise-free variables, *i.e*., without (or with ignorable) fluctuations, which implies that the covariance of any two participating reactants is zero (or close to zero).

Each of these assumptions constitutes a sufficient condition for the direct use of a rate function as the propensity function and applies, in principle, to GMA as well as other systems. The validity of these conditions will be discussed later. Specifically, the first condition will be addressed in the Results section under the headings "0^th^-order reaction kinetics" and "1^st^-order reaction kinetics, " while the second condition will be discussed under the heading "Real-valued order monomolecular reaction kinetics." The third condition will be the focus of Equations (29-36) and their associated explanations.

In reality, the rates of reactions in biochemical systems are commonly nonlinear functions of the reactant species, and fluctuations within each species are not necessarily ignorable. Therefore, to the valid use of an equation-based model in a stochastic simulation mandates that we know how to define a proper propensity function. The following section addresses this issue. It uses statistical techniques to characterize estimates for both the mean and variance of the propensity function, and these features will allow an assessment of the validity of the assumption *α*(**X**) = *f_s_*(**X**) and prescribe adjustments if the assumption is not valid.

## Methods

### Deriving the mean and variance of a power-law function of random variables

Consider a generic power-law function of random variables *X_s _*with the format $PL\left(X\right)=k\overset{N_{s}}{\underset{s=1}{\prod }}X_{s}^{f_{s}}$. Estimates of its mean *μ_PL _*and variance *σ_PL _*are given as

(8) μPL≈k∏s=1Nsμsfs exp∑i<jNsfifjcovlogXi,logXj

(9) $${\sigma_{PL}}^{2}\approx {\mu_{PL}}^{2}\Omega$$

(for details, see Additional file 1). Here,

(10) $$\Omega =\overset{N_{s}}{\underset{s=1}{\sum }}f_{s}\mu_{s}^{-2}\sigma_{s}^{2}+2\overset{N_{s}}{\underset{i<j}{\sum }}f_{i}f_{j}\;\text{cov}\left[1\text{og}X_{i},\log X_{j}\right]$$

and *μ_s _*= E[*X_s_*] and $\sigma_{s}^{2}=\text{E}\left[{\left(X_{s}-\mu_{s}\right)}^{2}\right]$ are the mean and variance of random variable *X_s_*, respectively. If we choose to express cov [log*X_i_*, log*X_j_*] as a function of *μ_s_*, *σ_s_*^2 ^and covariance *σ_ij _*= cov [*X_i_*, *X_j_*], using a Taylor approximation, we obtain

(11) μPL≈k∏s=1Nsμsfs exp-12∑s=1Nsfsσs2/μs2+12Ω

(12) $${\sigma_{PL}}^{2}\approx {\mu_{PL}}^{2}\Omega ,$$

where

(13) $$\begin{array}{c} \Omega \approx \overset{N_{s}}{\underset{s=1}{\sum }}f_{s}{\left(\sigma_{s}/\mu_{s}\right)}^{2}+2\overset{N_{s}}{\underset{i<j}{\sum }}f_{i}f_{j}\left\{\sigma_{ij}/\left(\mu_{i}\mu_{j}\right)\right)\\ +\frac{1}{2}\log\left(\mu_{i}\right){\left(\sigma_{j}/\mu_{j}\right)}^{2}+\frac{1}{2}\log\left(\mu_{j}\right){\left(\sigma_{i}/\mu_{i}\right)}^{2}\\ \left(-\frac{1}{4}{\left(\sigma_{i}/\mu_{i}\right)}^{2}{\left(\sigma_{j}/\mu_{j}\right)}^{2}\right\}. \end{array}$$

Since many biochemical variables approximately follow a log-normal distribution [20-22], it is valuable to consider the special situation where (*X*_1_, ..., *X_s_*)is log-normally distributed (*i.e*., (log*X*_1_, ..., log*X_s_*) is normally distributed). In such a case, a simpler alternative way to calculate cov [log*X_i_*, log*X_j_*] is

(14) $$\text{cov}\left[1\text{og}X_{i},\log X_{j}\right]=\log\left(1+\frac{\sigma_{ij}}{\mu_{i}\mu_{j}}\right).$$

[23]. By substituting this result into (8)-(10), one obtains

(15) $$\mu_{PL}\approx k\overset{N_{s}}{\underset{s=1}{\prod }}\mu_{s}^{f_{s}}\overset{N_{s}}{\underset{i<j}{\prod }}{\left(1+\frac{\sigma_{ij}}{\mu_{i}\mu_{j}}\right)}^{f_{i}f_{j}}$$

(16) $${\sigma_{PL}}^{2}\approx {\mu_{PL}}^{2}\Omega ,$$

where

(17) Ω= ∑s=1Nsfsσsμs2+2∑i<jNsfifj log1+σijμiμj.

The approximation formulae for *μ_PL _*and *σ_PL_*^2 ^in eqns. (8)-(10) provide an easy numerical implementation if observation data are available to estimate cov [log*X_i_*, log*X_j_*]. Furthermore, Equations (11)-(13) demonstrate how *μ_PL _*and *σ_PL_*^2 ^are related to *μ_s_*, *σ_s_*^2 ^and *σ_ij_*; however, the price of this insight is paid by the possible inaccuracy introduced through the Taylor approximation. Equations (15)-(17) also provide a functional dependence of *μ_PL _*and *σ_PL_*^2 ^on (*μ_s_*, *σ_s_*^2^, *σ_ij_*), but it is only valid if the additional assumption of log-normality is acceptable.

### Deriving proper propensity functions for stochastic simulations from differential equation-based models

Assuming that the GMA model faithfully captures the average behaviour of a biochemical reaction system and recalling $\left[X\left(t\right)\right]={\left(\left[X_{1}\left(t\right)\right],\ldots ,\left[X_{N_{s}}\left(t\right)\right]\right)}^{T}$, the expected metabolite numbers are defined as the expectation

(18) $$E\left[X\right]=\left[X\right]\Phi ,$$

where Φ is the system size as defined above.

To describe the reaction channel *R_r _*stochastically, one needs the state update vector **v**_*r *_and must characterize the quantity of molecules flowing through of reaction channel *R_r _*during a small time interval. The key concept of this type of description is the propensity function *α*_*r*_(**x**), which is defined as

(19) $$\begin{array}{c} \alpha_{r}\left(x\right)dt=\text{the}\;\text{probability}\;\text{that}\;\text{exactly}\;\text{one}\;\text{reaction}\\ R_{r}\;\text{will}\;\text{occur}\;\text{some}\;\text{where}\;\text{inside}\;U\;\text{within}\;\text{infinitesmal}\\ \text{interval}\;\left(t,t+dt\right),\;\text{given}\;\text{current}\;\text{state}\;X\left(t\right)=x. \end{array}$$

[1]. Because of the probabilistic nature of the propensity function, **X**(t) is no longer deterministic, and the result is instead stochastic and based on the transition probability

(20) $$P\left(x,t|x_{0},t_{0}\right)=\text{Prob}\left\{X\left(t\right)=x,\;\text{given}\;X\left(t_{0}\right)=x_{0}\right\},$$

which follows the chemical master equation (CME)

(21) $$\begin{array}{ccc} \frac{\partial P\left(x,t|x_{0},t_{0}\right)}{\partial t} & =\overset{N_{r}}{\underset{r=1}{\sum }}[\alpha_{r}\left(x+v_{r}\right)P\left(x+v_{r},t|x_{0},t_{0}\right) & \\ & \;-\alpha_{r}\left(x\right)P\left(x,t|x_{0},t_{0}\right)] \end{array}$$

Updating CME requires knowledge of every possible combination of all species counts within the population, which immediately implies that it can be solved analytically for only a few very simple systems and that numerical solutions are usually prohibitively expensive [24]. To address the inherent intractability of CME, Gillespie developed an algorithm, called the *Stochastic Simulation Algorithm *(SSA), to simulate CME models [1]. SSA is an exact procedure for numerically simulating the time evolution of a well-stirred reaction system. It is rigorously based on the same microphysical premise that underlies CME and gives a more realistic representation of a system's evolution than a deterministic reaction rate equation represented by ODEs. SSA requires knowledge of the propensity function, which however is truly available only for elementary reactions. These reactions include: 1) 0^th ^order reactions, exemplified with the generation of a molecule at a constant rate; 2) 1^st ^order monomolecular reactions, such as an elemental chemical conversion or decay of a single molecule; 3) 2^nd ^order bimolecular reactions, including reactive collisions between two molecules of the same or different species. The reactive collision of more than two molecules at exactly the same time is considered highly unlikely and modelled as two or more sequential bimolecular reactions.

For elementary reactions, the propensity function of reaction *R_r _*is computed as the product of a stochastic rate constant *c_r _*and the number *h_r _*of distinct combinations of reactant molecules, i.e.

(22) $$\alpha_{r}\left(x\right)=c_{r}h_{r}\left(x\right),\;r=1,\ldots ,N_{r}.$$

Here $h_{r}\left(x\right)=\left\{\begin{array}{c} \overset{N_{s}}{\underset{s=1}{\prod }}\left(\begin{array}{c} x_{s}\\ {\underset{-}{v}}_{rs} \end{array}\right)\approx \frac{\overset{N_{s}}{\underset{s=1}{\prod }}x_{s}^{{\underset{-}{v}}_{rs}}}{\overset{N_{s}}{\underset{s=1}{\prod }}{\underset{-}{v}}_{rs}!},\text{for}\;x_{s}\geq {\underset{-}{v}}_{rs}>0\\ 0,\;\text{otherwise} \end{array}\right)$, where *x_s _*is the sample value of random variable *X_s_*. The approximation is invoked when *x_s _*is large and (*x_s _*- 1), ..., (*x_s _*- *v_rs _*+ 1) are approximately equal to *x_s_*.

In Gillespie's original formulation [1]*c_r _*is a constant that only depends on the physical properties of the reactant molecules and the temperature of the system, and *c_r_dt *is the probability that a particular combination of reactant molecules will react within the next infinitesimally small time interval (*t*, *t *+ *dt*). The constant *c_r _*can be calculated from the corresponding deterministic rate constants, if they are known.

Since the assumption of mass action kinetics is not valid generally, especially in spatially restricted environments and in situations dominated by macromolecular crowding, we address the broader scenario where c_*r *_is not a constant but a function of the reactant concentrations. Thus, we denote c_*r *_as a *stochastic rate function*, while retaining the definition of *h_r _*as above. Knowing that any positive-valued differentiable function can be approximated locally by a power-law function, we assume the functional form of the stochastic rate function as

(23) $$c_{r}\left(x\right)=\kappa_{r}\overset{N_{s}}{\underset{s=1}{\prod }}x_{s}{\left(t\right)}^{\varepsilon_{rs}}.$$

Here, *κ_r _*and *ε_rs _*are constants that will be specified in the next section, and *r *= 1, ..., *N_r_*. Note that *ε_rs _*are now real-valued. Once the stochastic rate function is determined (see below), the propensity function can be calculated as

(24) $$\alpha_{r}\left(x\right)=c_{r}\left(x\right)h_{r}\left(x\right)=\frac{\kappa_{r}}{\overset{N_{s}}{\underset{s=1}{\prod }}{\underset{-}{v}}_{rs}!}\overset{N_{s}}{\underset{s=1}{\prod }}x_{s}^{{\underset{-}{v}}_{rs}+\varepsilon_{rs}}.$$

In order to identify the functional expression for a stochastic rate function, and thus the propensity function, we consider the connection between the stochastic and the deterministic equation models. By multiplying CME with **x **and summing over all **x**, we obtain

(25) $$\frac{d}{dt}E\left[X\left(t\right)\right]=\overset{N_{r}}{\underset{r=1}{\sum }}v_{r}E\left[\alpha_{r}\left(X\left(t\right)\right)\right].$$

Similarly, the expectation for any species *X_s_*(*t*) is given as

(26) $$\frac{d}{dt}E\left[X_{s}\left(t\right)\right]=\overset{N_{r}}{\underset{r=1}{\sum }}v_{rs}E\left[\alpha_{r}\left(X\left(t\right)\right)\right],\;s=1,\ldots ,\;N_{s}.$$

The details of these derivations are shown in Additional file 1.

We can use these results directly to compute the propensity function for a stochastic GMA model, assuming that its deterministic counterpart is well defined. Specifically, we start with the deterministic GMA equation for *X_s_*,

(27) $$\frac{d}{dt}\left[X_{s}\left(t\right)\right]=\overset{N_{r}}{\underset{r=1}{\sum }}v_{rs}k_{r}\overset{N_{s}}{\underset{s^{\prime }=1}{\prod }}{\left[X_{s^{\prime }}\left(t\right)\right]}^{f_{rs^{\prime }}},\;s=1,\ldots ,N_{s},$$

where *v_rs_*, *k_r _*and *f*_*rs*' _are again the stoichiometric coefficients, rate constants, and kinetic orders, respectively. By substituting $\left[X_{s}\right]=\frac{E\left[X_{s}\right]}{\Phi }$ from Equation (18) into this GMA model, we obtain a "particle-based" equation of the format

$$\frac{d}{dt}\left(\frac{E\left[X_{s}\right]}{\Phi }\right)=\overset{N_{r}}{\underset{r=1}{\sum }}v_{rs}k_{r}\overset{N_{s}}{\underset{s^{\prime }=1}{\prod }}{\left(\frac{E\left[X_{s^{\prime }}\right]}{\Phi }\right)}^{f_{rs^{\prime }}},s=1,\ldots ,N_{s}.$$

Elementary operations allow us to rewrite this equation as

(28) $$\frac{d}{dt}\left(E\left[X_{s}\right]\right)=\overset{N_{r}}{\underset{r=1}{\sum }}v_{rs}k_{r}\Phi^{1-F_{r}}\overset{N_{s}}{\underset{s^{\prime }=1}{\prod }}E{\left[X_{s^{\prime }}\right]}^{f_{rs^{\prime }}},\;s=1,\ldots ,N_{s},$$

where $F_{r}=\overset{N_{s}}{\underset{s^{\prime }=1}{\sum }}f_{rs^{\prime }}$. In this formulation, the differential operator is justified only when large numbers of molecules are involved. The assumption that the deterministic equations precisely capture the average behaviour of the biochemical reaction system directly equates the stochastic CME (25) to the deterministic equation based model (28)

(29) $$E\left[\alpha_{r}\left(X\left(t\right)\right)\right]=k_{r}\Phi^{1-F_{r}}\overset{N_{s}}{\underset{s^{\prime }=1}{\prod }}E{\left[X_{s^{\prime }}\right]}^{f_{rs^{\prime }}}.$$

Now we have two choices for approximating the expectation of the propensity function on left-hand side of equation (29):

1) adopt a zero-covariance assumption as was done in [25], which implies ignoring random fluctuations within every species as well as their correlations. This assumption is only justified for some special cases such as monomolecular and bimolecular reactions under the thermodynamic limit (cf. [4,6]), but is not necessary valid in generality. Here the thermodynamic limit is defined as a finite concentration limit which the system reaches when both population and volume approach infinity. Under this assumption, the left hand side of (29) becomes

(30) $$\begin{array}{ccc} E\left[\alpha_{r}\left(x\right)\right] & =E\left[\frac{\kappa_{r}}{\overset{N_{s}}{\underset{s=1}{\prod }}{\underset{-}{v}}_{rs}!}\overset{N_{s}}{\underset{s=1}{\prod }}x_{s}^{{\underset{-}{v}}_{rs}+\varepsilon_{rs}}\right] & \\ & =\frac{\kappa_{r}}{\overset{N_{s}}{\underset{s=1}{\prod }}{\underset{-}{v}}_{rs}!}\overset{N_{s}}{\underset{s=1}{\prod }}E{\left[X_{s}\right]}^{{\underset{-}{v}}_{rs}+\varepsilon_{rs}} \end{array}$$

for every *r *= 1, ..., *N_r_*, and Equation (24) yields

(31) $$\begin{array}{ccc} \varepsilon_{rs} & =f_{rs}-{\underset{-}{v}}_{rs} & \\ \kappa_{r} & =k_{r}\Phi^{1-F_{r}}\overset{N_{s}}{\underset{s=1}{\prod }}{\underset{-}{v}}_{rs}!\\ c_{r}\left(x\right) & =k_{r}\Phi^{1-F_{r}}\overset{N_{s}}{\underset{s=1}{\prod }}{\underset{-}{v}}_{rs}!x_{s}^{\varepsilon_{rs}} \end{array}$$

and

(32) $$\alpha_{r_{-}0}\left(x\right)=k_{r}\Phi^{1-F_{r}}\overset{N_{s}}{\underset{s=1}{\prod }}x_{s}^{f_{rs}}.$$

Here, the index *r*_0 is used to distinguish this 0-covariance propensity function from a second type of propensity in the next section.

With the zero-covariance assumption, one can substitute (32) back into the equation for the expectation for each species, which yields

(33) $$\frac{d}{dt}E\left[X_{s}\left(t\right)\right]=\overset{N_{r}}{\underset{r=1}{\sum }}v_{rs}k_{r}\Phi^{1-F_{s}}\overset{N_{s}}{\underset{s=1}{\prod }}\mu_{s}^{f_{rs}}$$

for every *s *= 1, ..., *N_s_*.. Note that this result is exactly equivalent to the equation-based model (27).

Equation (33) is based on assumption that both the fluctuations within species and their correlations are ignorable, which is not necessarily true in reality. If one uses it in simulations where the assumptions are not satisfied, it is possible that the means for the molecular species are significantly different from the corresponding equation-based model values. This discrepancy arises because the evolution of each species in the stochastic simulation is in truth affected by the covariance which is not necessarily zero, as it was assumed. This phenomenon was observed by Paulsson and collaborators [26] and further discussed in different moment-based approaches [6,7]. To assess the applicability limit of the propensity defined by (32), we can apply approximation techniques as shown in eqns. (8)-(10) on the functional expression of *α*_*r*_0 _and obtain mean and variance as

(34) $$\begin{array}{ccc} \mu_{\alpha_{r_{-}0}} & =E\left[\alpha_{r_{-}0}\left(X\left(t\right)\right)\right] & \\ & \approx k_{r}\Phi^{1-F_{r}}\overset{N_{s}}{\underset{s^{\prime }=1}{\prod }}E{\left[X_{s^{\prime }}\right]}^{f_{rs^{\prime }}}\exp\left(\overset{N_{s}}{\underset{i<j}{\sum }}f_{ri}f_{rj}\;\text{cov}\left[1\text{og}X_{i},\log X_{j}\right]\right) \end{array}$$

(35) $${\sigma_{\alpha_{r_{-}0}}}^{2}\approx {\mu_{\alpha_{r_{-}0}}}^{2}\Omega_{r},$$

where

(36) $$\Omega_{r}=\overset{N_{s}}{\underset{s=1}{\sum }}f_{rs}\mu_{s}^{-2}\sigma_{s}^{2}+2\overset{N_{s}}{\underset{i<j}{\sum }}f_{ri}f_{rj}\;\text{cov}\left[1\text{og}X_{i},\log X_{j}\right],$$

for every *s *= 1, ..., *N_s_*. These expressions demonstrate that even with large numbers of molecules the mean of CME does not always converge to the GMA model. Indeed, the convergence is only guaranteed in one of the following special situations: 1) the reaction is of 0^th ^order; 2) the reaction is a real value-order monomolecular reaction, with 1^st ^order reaction as a special case; 3) the covariance contribution in (34) is sufficiently small to be ignored for all participating reactant species of a particular reaction channel. Except for these three special situations, the covariance as shown in (34) significantly affects the mean dynamics. Therefore, stochastic simulations using zero-covariance propensity functions will in general yield means different from what the deterministic GMA model produces. How large these differences are cannot be said in generality. Under the assumption that the GMA model correctly captures the mean dynamics of every species, this conclusion means that *α*_*r*_0 _is not necessarily an accurate propensity function for stochastic simulations, and the direct conversion of the equation-based model into a propensity function must be considered with caution.

Moreover, there is no theoretical basis to assume that there are no fluctuations in the molecular species or that these are independent. Therefore, we need to consider the second treatment of the expectation of the propensity function and study the possible effects of a non-zero covariance.

2) We again assume that the GMA model is well defined, which implies that information regarding the species correlations and fluctuations has been captured in the parameters of the GMA model on the left hand size of Equations (7) and (28). To gain information regarding correlations, we use Taylor expansion to approximate the propensity function (see Additional file 1 for details):

(37) E[αr(X(t))]=E[κr∏s=1Nsv¯rs!∏s=1NsXsv¯rs+εrs]≈κr∏s=1Nsv¯rs!∏s=1NsE[Xs]v¯rs+εrs×exp(∑i<jNs(v¯ri+εri)(v¯rj+εrj) cov[1ogXi,logXj])

After substitution of (37) in (29), one obtains

$$\begin{array}{ccc} \kappa_{r} & =k_{r}\Phi^{1-F_{r}}\overset{N_{s}}{\underset{s=1}{\prod }}{\underset{-}{v}}_{rs}!\exp\left(-\overset{N_{s}}{\underset{i<j}{\sum }}f_{ri}f_{rj}\;\text{cov}\left[1\text{og}X_{i},\log X_{j}\right]\right) & \\ \varepsilon_{rs} & =f_{rs}-{\underset{-}{v}}_{rs}. \end{array}$$

Given the state **x **of the system at time *t*, the stochastic rate function of reaction *R_r _*is

(38) $$\begin{array}{ccc} c_{r}\left(x\right) & =\kappa_{r}\overset{N_{s}}{\underset{s=1}{\prod }}x_{s}^{\varepsilon_{rs}} & \\ & =k_{r}\Phi^{1-F_{r}}\overset{N_{s}}{\underset{s=1}{\prod }}{\underset{-}{v}}_{rs}!\\ & \times \exp\;\left(-\overset{N_{s}}{\underset{i<j}{\sum }}f_{ri}f_{rj}\;\text{cov}\left[1\text{og}X_{i}\left(t\right),\log X_{j}\left(t\right)\right]\right)\overset{N_{s}}{\underset{s=1}{\prod }}x_{s}^{f_{rs}-{\underset{-}{v}}_{rs}}. \end{array}$$

Here it is important to understand that although the random variables {*X_s_*}_*s*∈*S *_appear in the expression *c_r_*(**x**), *c_r_*(**x**) is not a function of random variables but a deterministic function. The reason is that the cov [log*X_i_*(*t*), log*X_j_*(*t*)] in the composition of *c_r_*(**x**), which as the numerical characteristic of the random variables {*X_s_*}_*s*∈*S*_, is deterministic. Therefore, the stochastic rate function *c_r_*(**x**) is a well-justified deterministic function that is affected by both the state of the system $\left[x_{1},\ldots ,x_{N_{s}}\right]$ and cov [log*X_i_*(*t*), log*X_j_*(*t*)], the numerical characteristic of fluctuations in the random variables {*X_s_*}_*s*∈*S*_.

Given the expression *c_r_*(**x**), the propensity function is

(39) $$\begin{array}{ccc} \alpha_{r}\left(x\right) & =c_{r}\left(x\right)h_{r}\left(x\right) & \\ & =k_{r}\Phi^{1-F_{r}}\overset{N_{s}}{\underset{s=1}{\prod }}x_{s}^{f_{rs}}\\ & \times \exp\;\left(-\overset{N_{s}}{\underset{i<j}{\sum }}f_{ri}f_{rj}\;\text{cov}\left[1\text{og}X_{i}\left(t\right),\log X_{j}\left(t\right)\right]\right). \end{array}$$

These results are based on the assumption that there are large numbers of molecules for all reactant species participating in reaction *R_r_*. For simplicity of discussion, we define the *propensity adjustment factor *(paf) of reaction *R_r _*as

(40) $$paf\left(t\right)≜\exp\left(-\overset{N_{s}}{\underset{i<j}{\sum }}f_{ri}f_{rj}\;\text{cov}\left[1\text{og}X_{i}\left(t\right),\log X_{j}\left(t\right)\right]\right).$$

*paf *is a function of time *t *and represents the contribution of the reactants to correlations among species in the calculation of the propensity function for reaction *R_r_*. We denote the propensity function in (39), which accounts for the contribution of the covariance, as *α*_*r*_cov_, in order to distinguish it from the propensity function *α*_*r*_0 _(32), which is based on the assumption of zero-covariance, *i.e*.,

(41) $$\alpha_{r_{-}\text{cov}}\left(x\right)=paf\left(t\right)k_{r}\Phi^{1-F_{r}}\overset{N_{s}}{\underset{s=1}{\prod }}x_{s}^{f_{rs}}.$$

Remembering that cov [log*X_i_*(*t*), log*X_j_*(*t*)], which is a component in both the stochastic rate function *c_r_*(**x**) and now in the function *paf*(*t*), is a deterministic function rather than a function of random variables, *paf*(*t*) is a deterministic correction to the kinetic constant *k_r _*in the construction of *α*_*r*_cov _in (41), which corrects the stochastic simulation toward the correct average.

In contrast to the propensity function *α*_*r*_0_, *α*_*r*_cov _leads to accurate stochastic simulations. To illustrate this difference, we analyze $\frac{d}{dt}E\left[X_{s}\left(t\right)\right]$ as follows: We apply the approximation techniques in eqns. (9)-(11) in order to obtain the mean and variance of the propensity function *α*_*r*_cov_:

(42) $$\mu_{\alpha_{r_{-}\text{cov}\;}}=E\left[\alpha_{r_{-}\text{cov}}\left(X\left(t\right)\right)\right]\approx k_{r}\Phi^{1-F_{r}}\overset{N_{s}}{\underset{s^{\prime }=1}{\prod }}E{\left[X_{s^{\prime }}\right]}^{f_{rs^{\prime }}}$$

(43) $${\sigma_{r_{-}\text{cov}\;}}^{2}\approx {\mu_{\alpha_{r_{-}\text{cov}\;}}}^{2}\Omega_{r}.$$

Here

(44) $$\Omega_{r}=\overset{N_{s}}{\underset{s=1}{\sum }}f_{rs}\mu_{s}^{-2}\sigma_{s}^{2}+2\overset{N_{s}}{\underset{i<j}{\sum }}f_{ri}f_{rj}\;\text{cov}\left[1\text{og}X_{i},\log X_{j}\right].$$

By substituting (42) back into the derivation of CME (26), one obtains

(45) $$\begin{array}{c} \frac{d}{dt}E\left[X_{s}\left(t\right)\right]\\ =\overset{N_{r}}{\underset{r=1}{\sum }}v_{rs}E\left[\alpha_{r_{-}\text{cov}}\left(X\left(t\right)\right)\right]\\ \approx \overset{N_{r}}{\underset{r=1}{\sum }}v_{rs}k_{r}\Phi^{1-F_{r}}\overset{N_{s}}{\underset{s^{\prime }=1}{\prod }}\mu_{s^{\prime }}^{f_{rs^{\prime }}} \end{array}$$

for every *s *= 1, ..., *N_s_*, which is equivalent in approximation to the GMA model (28). In the other words, the mean of every molecular species obtained by using *α*_*r*_cov _in the CME derived equation (27) is approximately identical to the corresponding macroscopic variable in the GMA model.

**Calculation of **cov [log*X_i_*(*t*), log*X_j_*(*t*)**]**

When data in the form of multiple time series for all the reactants are available, it is possible to compute cov [log*X_i_*(*t*), log*X_j_*(*t*)**] **directly from these data. Once this covariance is known, the function *paf*, *α*_*r*_cov _and the mean dynamics can all be assessed. Alas, the availability of several time series data for all reactants under comparable conditions is rare, so that cov [log*X_i_*(*t*), log*X_j_*(*t*)**] **must be estimated in a different manner.

If one can validly assume that the covariance based on *α*_*r*_0 _does not differ significantly from the covariance based on *α*_*r*_cov_, one may calculate cov [log*X_i_*(*t*), log*X_j_*(*t*)**] **by one of following methods.

Method 1:

One uses *α*_*r*_0 _to generate multiple sets of time series data of all reactants and then computes cov [log*X_i_*(*t*), log*X_j_*(*t*)**]**.

Method 2:

First, cov [log*X_i_*(*t*), log*X_j_*(*t*)**] **is expressed as a function of mean and covariance in one of the following ways; either as

(46) $$\begin{array}{c} \text{cov}\left[1\text{og}X_{i},\log X_{j}\right]\approx \sigma_{ij}/\left(\mu_{i}\mu_{j}\right)+\frac{1}{2}\log\left(\mu_{i}\right){\left(\sigma_{j}/\mu_{j}\right)}^{2}\\ +\frac{1}{2}\log\left(\mu_{j}\right){\left(\sigma_{i}/\mu_{i}\right)}^{2}-\frac{1}{4}{\left(\sigma_{i}/\mu_{i}\right)}^{2}{\left(\sigma_{j}/\mu_{j}\right)}^{2} \end{array}$$

or as Equation (14):

$$\text{cov}\left[1\text{og}X_{i},\log X_{j}\right]=\log\left(1+\frac{\sigma_{ij}}{\mu_{i}\mu_{j}}\right).$$

The first functional expression of cov [log*X_i_*(*t*), log*X_j_*(*t*)**] **is achieved by Taylor approximation, whereas the second expression is obtained by the additional assumption that the concentrations (*X*_1_, ..., *X_s_*) are log-normally distributed [8,23]. The consideration of a log-normal distribution is often justified by the fact that many biochemical data have indeed been observed to be log-normally distributed (*e.g*., [20-22]).

Second, one uses *α*_*r*_0 _to approximate the mean and covariance either by direct simulation, as shown in method 1, or by a moment-based approach, which is explained in Additional file 2, and which yields the differential equations

$$\begin{array}{ccc} \frac{\partial \mu_{s}}{\partial t} & \approx \overset{N_{r}}{\underset{r=1}{\sum }}v_{r,s}\left\{\alpha_{r_{-}0}\left(\mu \right)+\frac{1}{2}\overset{N_{s}}{\underset{m,n=1}{\sum }}\frac{\partial^{2}\alpha_{r\text{\_}0}\left(\mu \right)}{\partial X_{m}\partial X_{n}}\sigma_{mn}\right\} & \\ \frac{\partial \sigma_{ij}}{\partial t} & \approx \overset{N_{r}}{\underset{r=1}{\sum }}\left\{v_{r,i}\overset{N_{s}}{\underset{s=1}{\sum }}\frac{\partial \alpha_{r\text{\_}0}\left(\mu \right)}{\partial X_{s}}\sigma_{js}+v_{r,j}\overset{N_{s}}{\underset{s=1}{\sum }}\frac{\partial \alpha_{r\text{\_}0}\left(\mu \right)}{\partial X_{s}}\sigma_{is}\right)\\ & \;\left(+v_{r,i}v_{r,j}\left[\alpha_{r_{-}0}\left(\mu \right)+\frac{1}{2}\overset{N_{s}}{\underset{m,n=1}{\sum }}\frac{\partial^{2}\alpha_{r\text{\_}0}\left(\mu \right)}{\partial X_{m}\partial X_{n}}\sigma_{mn}\right]\right\} \end{array}$$

For convenience of computational implementation, the above equations can be written in matrix format

$$\begin{array}{ccc} \frac{\partial \mu }{\partial t} & \approx V^{T}\left(\alpha +\frac{1}{2}\alpha^{\prime \prime }⊙\sigma \right) & \\ \frac{\partial \sigma }{\partial t} & \approx {\left(\sigma \left(\alpha^{\prime }V\right)\right)}^{T}+\sigma \left(\alpha^{\prime }V\right)+V^{T}\Lambda V. \end{array}$$

Here for *r *= 1, ..., *N_r_*, and *s*, *m*, *n *= 1, ..., *N_s_*, $\mu ={\left(\mu_{1},\ldots ,\mu_{N_{s}}\right)}^{T}$, (*V*)_*rs *_= *v_rs_*, $\alpha ={\left(\alpha_{1},...,\alpha_{N_{r}}\right)}^{T}$, $\alpha^{\prime \prime }={\left(\alpha_{r}^{{}^{\prime \prime }},.\;.\;.\;,\alpha_{N_{r}}^{{}^{\prime \prime }}\right)}^{T}$, ${\left(\alpha_{r}^{{}^{\prime \prime }}\right)}_{mn}=\frac{\partial^{2}\alpha_{r}\left(X\right)}{\partial X_{m}\partial X_{n}}$, $\alpha_{r}^{{}^{\prime \prime }}⊙\sigma =\overset{N_{s}}{\underset{m,n=1}{\sum }}\frac{\partial^{2}\alpha_{r}\left(X\right)}{\partial X_{m}\partial X_{n}}{|}_{X=\mu }\sigma_{mn}$, $\alpha^{{}^{\prime \prime }}⊙\sigma ≜{\left(\alpha_{1}^{{}^{\prime \prime }}⊙\sigma ,\ldots ,\alpha_{N_{r}}^{{}^{\prime \prime }}⊙\sigma \right)}^{T}$, $\alpha^{\prime }=\left({\alpha_{1}}^{\prime },\ldots ,{\alpha_{N_{r}}}^{\prime }\right)$, ${\alpha_{r}}^{\prime }={\left(\frac{\partial \alpha_{r}\left(\mu \right)}{\partial X_{1}},\ldots ,\frac{\partial \alpha_{r}\left(\mu \right)}{\partial X_{N_{s}}}\right)}^{T}$, and Λ is a diagonal matrix with ${\left(\Lambda \right)}_{rr}=\alpha_{r}\left(\mu \right)+\frac{1}{2}\overset{N_{s}}{\underset{m,n=1}{\sum }}\frac{\partial^{2}\alpha_{r}\left(\mu \right)}{\partial X_{m}\partial X_{n}}\sigma_{mn}$.

### Statistical criteria for propensity adjustment

Suppose an equation-based model captures the average behavior of a stochastic system and one intends to find the propensity function for a stochastic simulation that will reproduce that means. One can use the 95% confidence interval to evaluate the need for a propensity adjustment. Specifically, for stable systems that will reach a steady state, we use the reversible reaction model as an example. If the steady state of the ODE *x_st _*is within the 95% confidence interval of *n *runs of stochastic simulations, i.e. $x_{st}\in \left[\mu_{st}-1.96\frac{\delta_{st}}{\sqrt{n}},\mu_{st}+1.96\frac{\delta_{st}}{\sqrt{n}}\right]$, then the rate function in the original ODEs can be used as the propensity without adjustment; otherwise propensity adjustment is needed. Here *μ_st _*and *δ_st _*can be attained from either a moment-base method or from *n *independent runs of stochastic simulations using propensity without adjustment. An example discussing a reversible reaction with feedback controls can be found in the results section.

For other systems that do not reach a steady state, but where instead transient characteristics are of the highest interest, one can judge the need of propensity adjustment by whether the pertinent characteristics of the ODEs are within the 95% confidence interval of the corresponding characteristic, which is given by a prediction from the moment-based method or from *n *runs of stochastic simulations. The Repressilator example in the result section will serve as a demonstration.

## Results

### Generic special cases

It is generally not valid to translate a rate from a deterministic biochemical model into a propensity function of the corresponding stochastic simulation without adjustment (see Equations. (34)-(36)). However, in some situations, the propensity adjustment (*e.g*., Equations (40)-(44)) is not needed, and in some other cases it becomes relatively simple.

1) 0^th^-order reaction kinetics

Consider a very simple equation-based model of the type

(47) $$\frac{d\left[X_{s}\left(t\right)\right]}{dt}=k_{r}\;\text{or}\;\frac{dE\left[X_{s}\left(t\right)\right]}{dt}=k_{r}\Phi ,$$

for all *s *= 1, ..., *N_s_*, *f_rs _*= 0. According to Equations (40)-(44), one obtains

$$\begin{array}{c} \Omega_{r}=0\\ {\sigma_{\alpha_{r}}}^{2}\approx 0\\ \mu_{\alpha_{r}}\approx \exp\left(\log\left(k_{r}\Phi \right)\right)=k_{r}\Phi \\ \text{i}\text{.e}\text{.}\;E\left[\alpha_{r}\left(X\right)\right]\approx \alpha_{r}\left(E\left[X\right]\right)\\ \alpha_{r\text{\_}\text{cov}\;}\approx k_{r}\Phi =\alpha_{r\text{\_}0}. \end{array}$$

Thus, for a 0^th^-order reaction, its rate equation can be taken directly as the propensity function in stochastic simulations.

2) 1^st^-order reaction kinetics

Direct application of Equations (40)-(44) yields

(48) $$\begin{array}{ccc} \frac{d\left[X_{i}\left(t\right)\right]}{dt} & =k_{r}\left[X_{j}\left(t\right)\right] & \\ \text{or}\;\frac{dE\left[X_{i}\left(t\right)\right]}{dt} & =k_{r}E\left[X_{j}\left(t\right)\right], \end{array}$$

*f_rs _*= *δ_sj_*, *i*, *j *= 1, ..., *N_s_*. Therefore, according to Equations (40)-(44)

$$\begin{array}{c} \Omega_{r}={\left(\sigma_{j}/\mu_{j}\right)}^{2}\\ {\left(\sigma_{\alpha_{r}}/\mu_{\alpha_{r}}\right)}^{2}={\left(\sigma_{j}/\mu_{j}\right)}^{2}\\ \mu_{\alpha_{r}}\approx \exp\left(\log\left(k_{r}\mu_{j}\right)\right)=k_{r}\mu_{j}\\ \text{i}\text{.e}\text{.}\;E\left[\alpha_{r}\left(X\right)\right]\approx \alpha_{r}\left(E\left[X\right]\right)\\ \alpha_{r_{-}\text{cov}}\left(X\right)\approx k_{r}X_{j}=\alpha_{r_{-}0}\left(X\right). \end{array}$$

Thus, for 1^st^-order reactions, the rate equation can again be taken directly as the propensity function in stochastic simulations.

3) Real-valued order monomolecular reaction kinetics

Consider a reaction with kinetics of the type

(49) $$\begin{array}{ccc} \frac{d\left[X_{i}\left(t\right)\right]}{dt} & =k_{r}{\left[X_{j}\left(t\right)\right]}^{f_{rj}} & \\ \text{or}\;\frac{dE\left[X_{i}\left(t\right)\right]}{dt} & =k_{r}\Phi^{1-f_{rj}}E{\left[X_{j}\left(t\right)\right]}^{f_{rj}}, \end{array}$$

*f_rj _*≠ 0, *f_rs _*= 0, for any *s *≠ *j*, *s *= 1, ..., *N_s_*. Equations (40)-(44) lead to

$$\begin{array}{c} \Omega_{r}={\left(\sigma_{j}/\mu_{j}\right)}^{2}\\ {\left(\sigma_{\alpha_{r}}/\mu_{\alpha_{r}}\right)}^{2}={\left(\sigma_{j}/\mu_{j}\right)}^{2}\\ \mu_{\alpha_{r}}\approx k_{r}\Phi^{1-f_{rj}}\mu_{j}^{f_{rj}}\;\text{i}\text{.e}\text{.}\;E\left[\alpha_{r}\left(X\right)\right]\approx \alpha_{r}\left(E\left[X\right]\right)\\ \alpha_{r_{-}\text{cov}}\left(X\right)\approx k_{r}\Phi^{1-f_{rj}}X_{j}^{f_{rj}}=\alpha_{r_{-}\text{0}}\left(X\right). \end{array}$$

Thus, for reaction kinetics involving a single variable and a real-valued order, the rate equation can again be taken as the propensity function in stochastic simulations.

4) 2^nd^-order reaction kinetics

This type of reaction can be expressed as

(50) $$\begin{array}{ccc} \frac{d}{dt}\left[X_{s}\left(t\right)\right] & =k_{r}\left[X_{i}\left(t\right)\right]\left[X_{j}\left(t\right)\right] & \\ \text{or}\;\frac{dE\left[X_{s}\left(t\right)\right]}{dt} & =k_{r}\Phi^{-1}E\left[X_{i}\left(t\right)\right]E\left[X_{j}\left(t\right)\right], \end{array}$$

*i*, *j *∈ {1, ..., *N_s_*}, *i *≠ *j*, *f_ri _*= *f_rj _*= 1, and *f_rs _*= 0, for all *s *≠ *i*, *j*. Therefore, according to Equations (40)-(44)

$$\begin{array}{ccc} \Omega_{r} & ={\left(\sigma_{i}/\mu_{i}\right)}^{2}+{\left(\sigma_{j}/\mu_{j}\right)}^{2}+2\text{cov}\left[1\text{og}X_{i},\log X_{j}\right] & \\ & ={\left(\sigma_{i}/\mu_{i}\right)}^{2}+{\left(\sigma_{s}/\mu_{s}\right)}^{2}+2\left\{\text{cov}\left[X_{i}/\mu_{i},X_{j}/\mu_{j}\right]\right)\\ & \left(+\frac{1}{2}\log\left(\mu_{i}\right){\left(\sigma_{j}/\mu_{j}\right)}^{2}+\frac{1}{2}\log\left(\mu_{j}\right){\left(\sigma_{i}/\mu_{i}\right)}^{2}-\frac{1}{4}{\left(\sigma_{i}/\mu_{i}\right)}^{2}{\left(\sigma_{j}/\mu_{j}\right)}^{2}\right\} \end{array}$$

σαr/μαr2=Ωr∂=σi/μi2+σj/μj2+2cov1ogXi,logXjμαr≈kr(NAV)-1μiμjαr-cov(X)=krΦ-1XiXj exp-cov1ogXi,logXj≠αr-0(X).

Thus, the proper propensity function for 2^nd^-order reactions is different from the rate equation. The difference can be ignored only if the contribution from the covariance is insignificant. In general, the rate equation yields only an approximate propensity function for stochastic simulations, and the approximation quality must be assessed on a case-by-case basis.

5) Bimolecular reaction with real-valued order kinetics

This type of reaction can be formulated as

(51) $$\begin{array}{ccc} \frac{d\left[X_{s}\left(t\right)\right]}{dt} & =k_{r}{\left[X_{i}\left(t\right)\right]}^{f_{i}}{\left[X_{j}\left(t\right)\right]}^{f_{j}} & \\ \text{or}\;\frac{dE\left[X_{s}\left(t\right)\right]}{dt} & =k_{r}\Phi^{1-f_{i}-f_{j}}E{\left[X_{i}\left(t\right)\right]}^{f_{i}}E{\left[X_{j}\left(t\right)\right]}^{f_{j}}, \end{array}$$

*i*, *j *∈ {1, ..., *N_s_*}, *i *≠ *j*, *f_ri_*, *f_rj _*≠ 0, and *f_rs _*= 0, for all *s *≠ *i*, *j*. According to Equations (40)-(44) we obtain

$$\begin{array}{ccc} \Omega_{r} & ={\left(\sigma_{i}/\mu_{i}\right)}^{2}+{\left(\sigma_{j}/\mu_{j}\right)}^{2}+2f_{i}f_{j}\;\text{cov}\left[1\text{og}X_{i},\log X_{j}\right] & \\ & ={\left(\sigma_{i}/\mu_{i}\right)}^{2}+{\left(\sigma_{j}/\mu_{j}\right)}^{2}+2f_{i}f_{j}\left\{\text{cov}\left[X_{i}/\mu_{i},X_{j}/\mu_{j}\right]\right)\\ & \left(+\frac{1}{2}\log\left(\mu_{i}\right){\left(\sigma_{j}/\mu_{j}\right)}^{2}+\frac{1}{2}\log\left(\mu_{j}\right){\left(\sigma_{i}/\mu_{i}\right)}^{2}-\frac{1}{4}{\left(\sigma_{i}/\mu_{i}\right)}^{2}{\left(\sigma_{j}/\mu_{j}\right)}^{2}\right\} \end{array}$$

σαr/μαr2=Ωr=σi/μi2+σj/μj2+2fifjcov1ogXi,logXjμαr≈krΦfi+fj-1μifiμjfjαr-cov(X)=krΦfi+fj-1XifiXjfj exp-fifjcov1ogXi,logXj≠αr-0(X).

For bimolecular reactions of complex order, the propensity function is different from the rate equation. The difference can be ignored only if the contribution from the covariance is insignificant.

### Power-law representation of a reversible reaction with feedback controls

**Figure 1:**
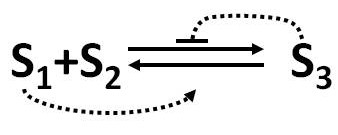
**Scheme of reversible reaction with feedback controls**. S_3 _inhibits the forward reaction and S_1 _activates the reverse reaction.

We consider a reversible reaction with feedback controls (see Figure 1) whose average behaviour is accurately described by the following GMA model

(52) $$\begin{array}{c} \frac{dx_{1}}{dt}=\frac{dx_{2}}{dt}=-\frac{dx_{3}}{dt}\\ =-k_{f}\Phi^{1-f_{1}-f_{2}-f_{3}}x_{1}^{f_{1}}x_{2}^{f_{2}}x_{3}^{f_{3}}+k_{b}\Phi^{1-g_{1}-g_{3}}x_{1}^{g_{1}}x_{3}^{g_{3}}. \end{array}$$

Here *S*_3 _feeds back to inhibit the forward reaction and *S*_1 _feeds back on the reverse reaction and accelerates it. The task is to develop a stochastic model whose performance converges to that of the deterministic GMA model. We can see from equations (52) that three variables *x*_1_, *x*_2 _and *x*_3 _contribute to the forward flux $k_{f}\Phi^{1-f_{1}-f_{2}-f_{3}}x_{1}^{f_{1}}x_{2}^{f_{2}}x_{3}^{f_{3}}$ and two variables *x*_1 _and *x*_3 _contribute to the backward flux $k_{b}\Phi^{1-g_{1}-g_{3}}x_{1}^{g_{1}}x_{3}^{g_{3}}$. Because several variables are involved, their covariance has the potential of affecting the forward and the backward propensity functions in a stochastic simulation. To obtain the covariance information, we formulate the moment equations (53) from the ODE model (52).

To simplify the calculation, as explained in detail in Additional file 2, we set the third central moment to zero and obtain a closed-form set of ODEs. Expressed differently, the rate of change in mean and covariance depends only on the functions of mean and covariance themselves, but not on higher-order moments. Thus,

(53) $$\begin{array}{ccc} \frac{\partial \mu }{\partial t} & \approx V^{T}\left(\alpha +\frac{1}{2}\alpha^{\prime \prime }⊙\sigma \right) & \\ \frac{\partial \sigma }{\partial t} & \approx {\left(\sigma \left(\alpha^{\prime }V\right)\right)}^{T}+\sigma \left(\alpha^{\prime }V\right)+V^{T}\Lambda V. \end{array}$$

Here *μ *= (*μ*_1_, *μ*_2_, *μ*_3_)*^T^*, $V=\left[\begin{array}{ccc} -1 & -1 & 1\\ 1 & 1 & -1 \end{array}\right]$, $\alpha ={\left(\alpha_{1},\alpha_{2}\right)}^{T}=\left[\begin{array}{c} k_{f}\Phi^{1-f_{1}-f_{2}-f_{3}}x_{1}^{f_{1}}x_{2}^{f_{2}}x_{3}^{f_{3}}\\ k_{b}\Phi^{1-g_{1}-g_{3}}x_{1}^{g_{1}}x_{3}^{g_{3}} \end{array}\right]$.

Moreover, for *r *= 1, 2 and *m*, *n *= 1, 2, 3, ${\left(\alpha_{r}^{{}^{\prime \prime }}\right)}_{mn}=\frac{\partial^{2}\alpha_{r}\left(X\right)}{\partial x_{m}\partial x_{n}}$, *α*" = (*α*_1_", *α*_2_")*^T^*, $\sigma =\left[\begin{array}{ccc} \sigma_{11} & \sigma_{12} & \sigma_{13}\\ \sigma_{21} & \sigma_{22} & \sigma_{23}\\ \sigma_{31} & \sigma_{32} & \sigma_{33} \end{array}\right]$, $\alpha_{r}^{{}^{\prime \prime }}⊙\sigma ≜\overset{3}{\underset{m,n=1}{\sum }}\frac{\partial^{2}\alpha_{r}\left(X\right)}{\partial x_{m}\partial x_{n}}{|}_{X=\mu }\sigma_{mn}$, *α*"⊙*σ *≜ (*α*_1_"⊙ *σ*, *α*_2_"⊙ *σ*)*^T^*, *α*' = (*α*_1_', *α*_2_'), ${\alpha_{r}}^{\prime }={\left(\frac{\partial \alpha_{r}\left(\mu \right)}{\partial x_{1}},\frac{\partial \alpha_{r}\left(\mu \right)}{\partial x_{2}},\frac{\partial \alpha_{r}\left(\mu \right)}{\partial x_{3}}\right)}^{T}$, and $\Lambda =\left[\begin{array}{cc} \alpha_{1}\left(\mu \right)+\frac{1}{2}\overset{3}{\underset{m,n=1}{\sum }}\frac{\partial^{2}\alpha_{1}\left(\mu \right)}{\partial x_{m}\partial x_{n}}\sigma_{mn} & 0\\ 0 & \alpha_{2}\left(\mu \right)+\frac{1}{2}\overset{3}{\underset{m,n=1}{\sum }}\frac{\partial^{2}\alpha_{2}\left(\mu \right)}{\partial x_{m}\partial x_{n}}\sigma_{mn} \end{array}\right]$.

**Figure 2:**
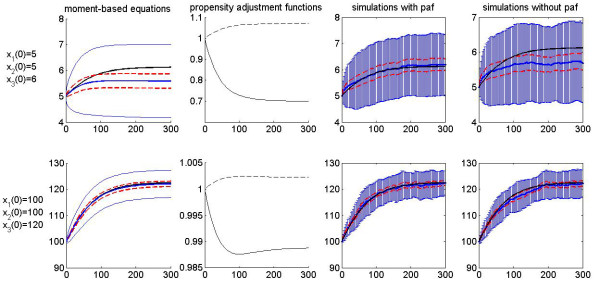
**Comparative simulation results for a reversible reaction with feedback controls**. In all panels, the *x*-axis denotes time in seconds and the *y*-axis represents the number of molecules of species S_1_. The upper and lower panels use two different sets of initial numbers of molecules, namely: (*x*_1_(0), *x*_2_(0), *x*_3_(0), *U*) = (5, 5, 6, 1*μm*^3^) and (*x*_1_(0), *x*_2_(0), *x*_3_(0), *U*) = (100, 100, 120, 20*μm*^3^), respectively. Other simulation parameters are (*f*_1_, *f*_2_, *f*_3_, *g*_1_, *g*_3_, *k_f_*, *k_g_*) = (1.3, 1.8, -1, 1, 1, 0.5, 0.5). In both the upper and lower panels, the first column compares the time evolution of *S*_1 _molecules by different methods: the black line shows the ODE solution of Equation (52) for *x*_1 _; the blue lines are the solutions of Equation (53) for *μ*_1 _and for *μ*_1 _± *σ*_1_, respectively. The red dotted lines framing the mean indicate the 95% confidence interval. The second column shows the propensity adjustment functions for the forward reaction (solid line) and the backward reaction (dashed line). The third column shows 100 independent stochastic simulations with propensity adjustment (blue means and error bars), in comparison with the ODE (Equation (52)) prediction (black line). The fourth column shows a second set of 100 independent stochastic simulations without propensity adjustment (blue means and error bars), in comparison with the ODE (Equation (52)) prediction (black line). The red dotted lines framing the mean in columns 3 and 4 again indicate the 95% confidence intervals.

Two initial conditions are chosen for representative simulations; they differ by a factor of 20 in species populations and reaction volume between the upper and lower panels of Figure 2. The purpose is to observe the thermodynamic limit of the systems: both scenarios have the same initial concentrations, but the system in the lower panel case has a larger species populations and reaction volume and can thus be regarded as the thermodynamic limit sample of system in the upper panel. As demonstrated by the figures in the first column, the moment approach predicts that for both population sizes the average trajectories of the stochastic model (without propensity adjustment) dynamics is lower than that of the equation-based model: the differences are about 10% of the steady-state value of the equation-based model in the upper figure and 1% in the lower figure; for 100 runs of the stochastic simulation, the steady-state value of the equation-based model lies outside the 95% confidence interval in the upper figure, while it is inside the interval in the lower figure. Therefore, we can expect that the propensity adjustment will significantly contribute to the stochastic simulation for the upper case while not for the lower case. This expectation is confirmed by the simulation results in the third and fourth columns. With the common assumption that the deterministic equations precisely capture the system's average behaviour, the case in the upper panel represents the situation where propensity adjustment is needed, while the lower panel represents the situation that a propensity without adjustment is sufficient when the system approaches its thermodynamics limit. This example furthermore demonstrates that either the moment approach or the stochastic simulations without propensity adjustment can be used to estimate whether there is a need to construct a propensity adjustment function for stochastic simulations.

### Repressilator

**Figure 3:**
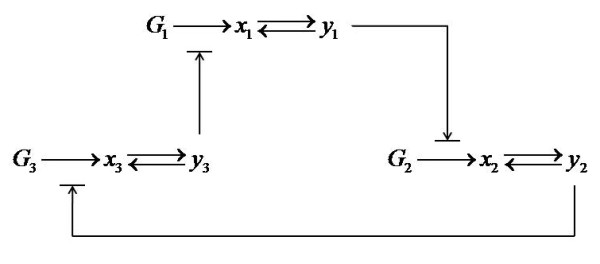
**Reaction scheme of the Repressilator**. Gene *G*_1 _codes for protein *x*_1_, whose dimer *y*_1 _represses the transcription of gene *G*_2_. Similarly, *y*_2_, the dimer of gene *G*_2_'s protein product *x*_2_, represses the transcription of gene *G*_3_, and *y*_3_, the dimer of gene *G*_3_'s protein product *x*_3_, represses the transcription of gene *G*_1_.

Interestingly, a propensity function may even be obtained through power-law approximation of some function that describes complex transient behaviours of a reaction network. As an example, consider the so-called *Repressilator *[27], which is a three-component genetic circuit where each component represses its downstream neighbour. More specifically (as shown in Figure 3), gene *G*_1 _codes for protein *x*_1_, whose dimer *y*_1 _subsequently represses the transcription of the gene *G*_2_. Similarly, *y*_2_, the dimer of gene *G*_2_'s protein product *x*_2_, represses the transcription of gene *G*_3_, and *y*_3_, the dimer of gene *G*_3_'s protein product *x*_3_, represses the transcription of gene *G*_1_. The corresponding differential equation model following mass action kinetics is given by [28]

(54) $$\begin{array}{ccc} {x_{i}}^{\prime } & =-2\kappa_{+}x_{i}^{2}+2\kappa_{-}y_{i}+\sigma m_{i}-\gamma_{p}x_{i} & \\ {y_{i}}^{\prime } & =\kappa_{+}x_{i}^{2}-\kappa_{-}y_{i}-k_{+}y_{i}d_{0,j}+k_{-}d_{r,j}\\ {d_{0,i}}^{\prime } & =-k_{+}y_{k}d_{0,i}+k_{-}d_{r,i}\\ {d_{r,i}}^{\prime } & =k_{+}y_{k}d_{0,i}-k_{-}d_{r,i}\\ {m_{i}}^{\prime } & =d_{0,i}-\gamma_{m}m_{i}, \end{array}$$

where *i *= 1, 2, 3; *j *= 2, 3, 1; *k *= 3, 1, 2; the rate constants are explained in the diagram below

$$\begin{array}{c} x_{i}+x_{i}\overset{\kappa_{+}}{\underset{\kappa_{-}}{⇌}}y_{i}\\ d_{0,i}+y_{k}\overset{\kappa_{+}}{\underset{\kappa_{-}}{⇌}}d_{r,i}\\ d_{0,i}\overset{\alpha }{\underset{}{\to }}d_{0,i}+m_{i}\\ m_{i}\overset{\alpha }{\underset{}{\to }}m_{i}+x_{i}\\ x_{i}\overset{\gamma_{p}}{\underset{}{\to }}\phi \\ m_{i}\overset{\gamma_{m}}{\underset{}{\to }}\phi \end{array}$$

Assuming that the reversible dimerization and the dissociation/association of a protein dimer from/to the promoter are much faster than other processes, the full systems can be reduced to

(55) $$\begin{array}{ccc} {x_{i}}^{\prime } & =\sigma p{\left(x_{i}\right)}^{-1}m_{i}-\gamma_{p}p{\left(x_{i}\right)}^{-1}x_{i} & \\ {m_{i}}^{\prime } & =\frac{\alpha d}{1+c_{d}c_{p}x_{k}^{2}}-\gamma_{m}m_{i} \end{array}$$

[28]. Here Φ = 1, $p\left(x_{i}\right)=1+4c_{p}x_{i}+\frac{4c_{d}c_{p}dx_{i}}{{\left(1+c_{d}c_{p}x_{i}^{2}\right)}^{2}}$, *c_p _*= *κ*_+_/*κ*_-_, *c_d _*= *k*_+_/*k*_- _and *d *= *d*_0, *i *_+ *d_r, i _*for *i *= 1, 2, 3. It has been shown that the simplified ODEs rather accurately approximate the transient dynamics of the full system by retaining the original oscillation period and amplitude.

In [28], the system (55) is further rescaled by setting $\tilde{t}=\gamma_{m}t,\;{\tilde{x}}_{i}=\sqrt{c_{d}c_{p}}x_{i}$ and $\tilde{m_{i}}=\left(\sigma \sqrt{c_{d}c_{p}}m_{i}\right)/\left(\gamma_{m}\beta \right)$, which yields

(56) $$\begin{array}{ccc} \frac{d{\tilde{x}}_{i}}{d\tilde{t}} & =\beta p{\left({\tilde{x}}_{i}\right)}^{-1}\tilde{m_{i}}-\beta p{\left({\tilde{x}}_{i}\right)}^{-1}{\tilde{x}}_{i} & \\ \frac{d\tilde{m_{i}}}{d\tilde{t}} & =\frac{\kappa d^{\prime }}{1+{\tilde{x}}_{k}^{2}}-\tilde{m_{i}}. \end{array}$$

**Figure 4:**
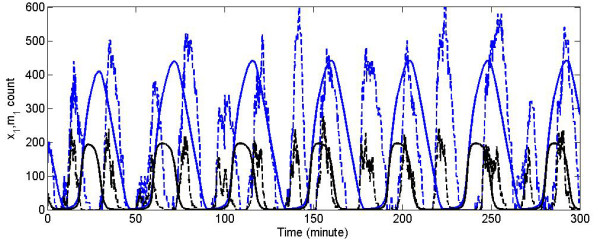
**Scaling of the Repressilator equations changes the oscillation period in the stochastic simulation**. Solid lines represent solutions of ODEs (56), while dotted lines are trajectories of a stochastic simulation; blue lines represent *x*_1 _and black lines represent *m*_1_.

Intriguingly, one makes the following observation. The scaled ODE system (56) is consistent with the original system (55) in oscillation amplitude and period. However, its corresponding stochastic model produces results that deviate substantially from the average responses. To see the effects of the transition from a deterministic to a stochastic model, we apply SSA to the scaled system (56). The main result is that the oscillation periods of both *x_i _*and *m_i _*are reduced to half (Figure 4). The reason is that, in the stochastic simulation, the oscillation period is very sensitive to the ratio of *x_i _*and *m_i_*, which has been altered by the scaling operation. Therefore, in general one needs to pay attention to how scaling may affect the stochastic performance when the model is generated through the conversion of an ODE model.

We can see from equations (55) that two variables *x_i _*and *m_i _*contribute to the production of *x_i_*; hence, their covariance could affect the propensity function of *x_i _*in the production reaction of a stochastic simulation. Similar to the example of a reversible reaction (Equation 52), it is therefore necessary to evaluate covariance effects and to judge whether the propensity function needs adjusting. Thus, we need to compare the difference between the dynamics of the phenomenological model (55) and the dynamics under the influence of covariance, which can be produced by either stochastic simulation or the moment approach.

**Figure 5:**
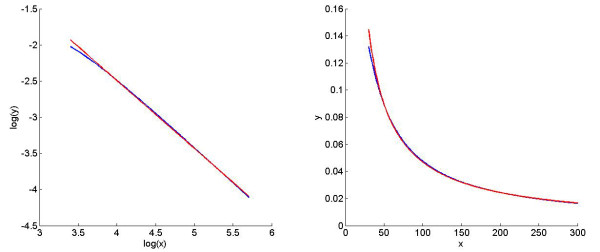
**Power-law approximation of *p*(*x_i_*)^-1^**. Left panel: Approximation of *y_i _*= *p*(*x_i_*)^-1 ^by a straight line in log-log space. Right panel: Corresponding power-law function in Cartesian space. Both axes are unitless.

The influence of the covariance on the dynamics of the stochastic simulation is relatively easy to assess: we simply use the terms on the right-hand side of the differential equations (54) as the propensity functions in SSA and obtain simulation results shown in the 2^nd ^and the 4^th ^panels of Figure 5. Obtaining the covariance-influenced dynamics with the moment-based approach is more complicated, and we need to discuss some implementation issues.

First, the moment-based approach requires information regarding the first and the second derivatives of *p*(*x_i_*)^-1^, which have rather complicated functional forms. To simplify the calculation, we replace the function *p*(*x_i_*)^-1 ^with an approximating power-law function. Specifically, suppose the original parameter values are *κ*_+ _= *k*_+ _= 5, *κ*_- _= *k*_- _= 100 and *d *= 20. Plotting the data (*x_i_*, *p*(*x_i_*)^-1^)in log-log space (Figure 5) indicates that the original function is represented well by a straight line:

$$\log y_{i}=\log3.5188-0.9384\log x_{i}.$$

for *x_i _*∈ [30, 300]. In Cartesian space, this line corresponds to the power-law function

$$y_{i}=3.5188x_{i}^{-0.9384},$$

which models the original function very well (see Figure 5). For *x_i _*∈ [1,30], this power-law function does not fit the original function precisely; the effect of this imprecision can be evaluated later at after we use this power-law function in the moment-based method. Moreover, using the truncated moment equations to estimate the mean and variance involves multiple approximations: First, the function *p *(*x_i_*)^-1 ^on the right-hand side of (55) is replaced by a power-law function (see Figure 5). Second, the result is approximated by Taylor expansion to the second order. Third, similar to the example of a reversible reaction, the central moment of the third degree is assumed to be zero, which leads to a closed-form ODE for the first two moments.

**Figure 6:**
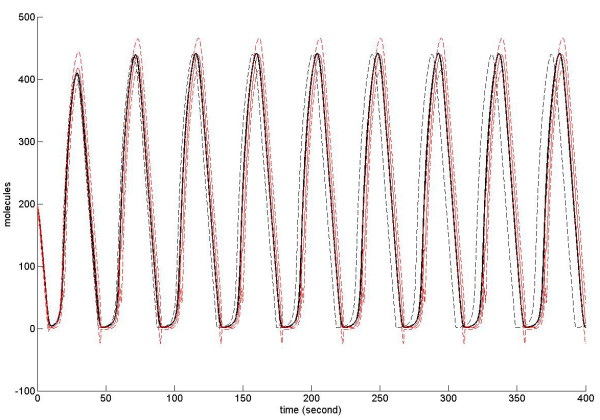
**Comparison of the dynamics of the Repressilator models using the original ODEs (55), the GMA approximation, and the moment approach based on the GMA approximation**. The mean of the moment approach based on the GMA approximation fits the original ODEs (55) very well up to about *t *= 400 s. Black bold line: solution of the original ODEs (55); black dashed line: the GMA approximation; Red line: mean of the moment approach based on the GMA approximation; red dashed lines framing around the red line: mean ± standard deviation, which were produced with the moment approach. *x*-axis is time in second, *y*-axis is the number of *x*_1 _molecules (unitless).

Solving the technical issues as described, one obtains the corresponding moment-based model of (55) (not shown) with results shown in Figure 6. Suppose one is particularly interested in the period and amplitude of the oscillation within a time interval between 0 and 400 seconds. As shown in Figure 6, the GMA approximation (black dashed line) fits the original ODEs (55) (bold black solid line) very well at the beginning, but as time goes on, the approximation error accumulates. As seen in the time interval [350, 400], the GMA approximation deviates from the original ODEs significantly. However, this does not mean that the GMA approximation cannot be used as a propensity function for stochastic simulations; the moment-based method with the GMA approximation shows that, when the GMA approximation is used as propensity function (without adjustment) for stochastic simulations, the resulting mean (red solid line) consistently fits the trajectory of the original ODEs (bold solid black line) very well up to about *t *= 400 seconds. The oscillation period and amplitude in the stochastic simulation based on the GMA approximation (without adjustment) are almost identical to those of the original ODEs. Therefore, a propensity adjustment for the GMA approximation is not needed, and the GMA approximation can be used as a propensity function for stochastic simulations. In other words, a stochastic model for the Repressilator system can be generated by using the scheme in (32) without propensity adjustment. Moreover, the imprecision caused by the power-law approximation can be tolerated when its corresponding moment-based mean matches the original ODEs sufficiently well with respect to the features of highest interest.

### Enzymatic reaction using a quasi-steady state assumption (QSSA)

We consider an enzymatic reaction following the Michaelis-Menten mechanism:

$$E+S\overset{k_{1}}{\underset{k_{-1}}{⇌}}ES\overset{k_{2}}{\underset{}{\to }}P+E.$$

Here enzyme E reacts with substrate S through a reversible reaction to form complex ES, which can proceed to yield product P and to release the enzyme E. By assuming the law of mass action for the reaction kinetics we obtain a set of differential equations for the system dynamics:

(57) $$\begin{array}{ccc} \frac{d\left[S\right]}{dt} & =k_{-1}\left[ES\right]-k_{1}\left[S\right]\left({\left[E\right]}_{0}-\left[ES\right]\right) & \\ \frac{d\left[ES\right]}{dt} & =k_{1}\left[S\right]\left({\left[E\right]}_{0}-\left[ES\right]\right)-\left(k_{1}+k_{2}\right)\left[ES\right]\\ \frac{d\left[P\right]}{dt} & =k_{2}\;\left[ES\right], \end{array}$$

where the total amount of enzyme in the form of free enzyme and complex [*E*]_0 _≜ [*E*] + [*ES*] is assumed to be constant. In addiction, by making the so-called *quasi steady state assumption *(QSSA) [29,30], assuming that the complex ES is essentially in steady state, we can assert $\frac{d\left[ES\right]}{dt}\approx 0$. As it has been discussed many times in the literature, QSSA reduces the system and leads to the approximate form

(58) $$\begin{array}{ccc} \frac{d\left[S\right]}{dt} & =-\frac{V_{\max}\left[S\right]}{K_{m}+\left[S\right]} & \\ \frac{d\left[P\right]}{dt} & =\frac{V_{\max}\left[S\right]}{K_{m}+\left[S\right]}, \end{array}$$

which is known as *Michaelis-Menten kinetics *[30]. The characterizing parameters are *V*_max _= *k*_2_[*E*]_0 _and *K_m _*= (*k*_-1 _+ *k*_2_)/*k*_1_.

Applying QSSA, Rao and Arkin [2] were able to reduce the CME of S and ES to a CME only containing S. For the reduced CME, the propensity function for the overall reaction *S *→ *P *is

(59) $$\alpha \left(s\right)=\frac{V_{\max}s}{K_{m}+s},$$

where the volume was scaled so that Φ = 1 and the lower-case letter *s *denotes the molecule count of species S. Instead of reviewing the relatively complicated manipulations with CME, we show in the following that the techniques described above lead directly from the equation-based model to the propensity function for the reduced system.

First, we recast the equation-based model into the GMA format [31], by introducing an auxiliary variable [*T*] ≜ *K_m _*+ [*S*]. The result,

(60) $$\begin{array}{ccc} \frac{d\left[S\right]}{dt} & =-V_{\max}\left[S\right]{\left[T\right]}^{-1} & \\ \frac{d\left[T\right]}{dt} & =\frac{d\left[S\right]}{dt}=-V_{\max}\left[S\right]{\left[T\right]}^{-1} \end{array}$$

is exactly equivalent to the reduced system in (58) with the initial condition [*S*]_0 _and [*T*]_0 _= *K_m _*+ [*S*]_0_. The corresponding stochastic model has only one reaction channel and the propensity function is

(61) $$\alpha \left(s,t\right)=V_{\max}st^{-1}.$$

The propensity adjustment factor can be set to 1 because *T *is a function of *s *and its covariance with *s *is therefore 1. By applying *t *= *K_m _*+ *s*, the propensity function can be simplified as

(62) α(s,t)=Vmaxst-1=V
maxs(Km+s)-1=α(s).

Thus, we arrive at the propensity function for the reduced system, which is identical to the result of Rao and Arkin obtained through manipulations of CME.

In the above derivation, we used the simplest type of recasting, where a new, auxiliary variable simply consists of an old variable plus a constant. This reformulation of the Michaelis-Menten process as a pair of GMA equations is a special case of a much more general recasting technique that permits the equivalent conversion of any system of ordinary differential equations into a power-law format [31]. However, this equivalence transformation imposes constraints on the variables of the GMA equations, and it is at this point unclear whether there are mathematical warranties ensuring that the proposed transition from differential to stochastic equations in general preserves these constraints in all cases. This question will require further investigation.

### Stochastic Focusing

Stochastic focusing [26] describes the phenomenon that the fluctuations of a chemical species can drive the system to reach a different steady state than what a deterministic ODE model predicts. To demonstrate the utility of propensity adjustment, we derive a stochastic model which produces consistent results with those of the deterministic model.

Following [32], we consider the following reactions system

(63) $$\begin{array}{c} \phi \overset{k_{1}}{\underset{}{\to }}I\overset{k_{2}}{\underset{}{\to }}P\overset{k_{3}}{\underset{}{\to }}\phi \\ I+S\overset{k_{4}}{\underset{}{\to }}S\\ S\overset{k_{6}}{\underset{k_{5}}{⇌}}\phi \end{array}$$

This system can be interpreted as follows: the intermediate species I is produced at constant rate *k*_1 _from some source *Φ *and degrades with rate *k*_4 _through the catalysis with signalling molecule S; the end product P is converted from species I at rate *k*_2 _and degrades at rate *k*_3_; the signalling molecule S is produced and degrades at rates *k*_5 _and *k*_6_, respectively. Moreover, the value of *k*_5 _is reduced to half at a certain time point to achieve a significant divergence effect. In order to capture the average dynamics of the system accurately, we use a power-law model in GMA format instead of the mass action rate law in [32].

(64) $$\begin{array}{ccc} \frac{di}{dt} & =k_{1}-k_{2}i-k_{4}i^{f_{I}}s^{f_{S}} & \\ \frac{dp}{dt} & =k_{2}i-k_{3}p\\ \frac{ds}{dt} & =k_{5}-k_{6}s \end{array}$$

The system size is set to 1. We can see from equations (64) that two variables *i *and *s *contribute to the degradation of *I *and that their covariance could therefore affect the propensity function of *I *in the degradation reaction of a stochastic simulation. To calculate the propensity adjustment function *paf*_4_(*t*) = exp (-*f_I _f_S _*cov [log *I*(*t*), log *S*(*t*)]) for reaction $R_{4}:I+S\overset{k_{4}}{\underset{}{\to }}S$, we formulate equations (*cf*. (60)) for the moments as

(65) $$\begin{array}{ccc} \frac{\partial \mu }{\partial t} & \approx V^{T}\left(\alpha +\frac{1}{2}\alpha^{\prime \prime }⊙\sigma \right) & \\ \frac{\partial \sigma }{\partial t} & \approx {\left(\sigma \left(\alpha^{\prime }V\right)\right)}^{T}+\sigma \left(\alpha^{\prime }V\right)+V^{T}\Lambda V. \end{array}$$

Here *μ *= (*μ_I_*, *μ_P_*, *μ_S_*)*^T^*, $V=\left[\begin{array}{ccc} 1 & 0 & 0\\ -1 & 1 & 0\\ 0 & -1 & 0\\ -1 & 0 & 0\\ 0 & 0 & 1\\ 0 & 0 & -1 \end{array}\right]$, $\alpha =\left[\begin{array}{c} \alpha_{1}\\ \alpha_{2}\\ \alpha_{3}\\ \alpha_{4}\\ \alpha_{5}\\ \alpha_{6} \end{array}\right]=\left[\begin{array}{c} k_{1}\\ k_{2}i\\ k_{3}p\\ k_{4}i^{f_{I}}s^{f_{S}}\\ k_{5}\\ k_{6}s \end{array}\right]$.

Moreover, for *r *= 1, ..., 6 and *m*, *n *= *i*, *p*, *s*, ${\left(\alpha_{r}^{{}^{\prime \prime }}\right)}_{mn}=\frac{\partial^{2}\alpha_{r}}{\partial m\partial n}$, *α*" = (*α*_1_", ..., *α*_6_")*^T^*, $\sigma =\left[\begin{array}{ccc} \sigma_{11} & \sigma_{12} & \sigma_{13}\\ \sigma_{21} & \sigma_{22} & \sigma_{23}\\ \sigma_{31} & \sigma_{32} & \sigma_{33} \end{array}\right]$, $\alpha_{r}^{{}^{\prime \prime }}⊙\sigma =\underset{m,n=i,p,s}{\sum }\frac{\partial^{2}\alpha_{r}\left(\mu \right)}{\partial m\partial n}\sigma_{mn}$, *α*"⊙ *σ *≜ (*α*_1 _"⊙ *σ*, ..., *α*_6_"⊙ *σ*)*^T^*, *α*' = (*α*_1_', ..., *α*_6_'), ${\alpha_{r}}^{\prime }={\left(\frac{\partial \alpha_{r}}{\partial i},\frac{\partial \alpha_{r}}{\partial p},\frac{\partial \alpha_{r}}{\partial s}\right)}^{T}$, and the diagonal matrix Λ is defined by ${\left(\Lambda \right)}_{rr}=\alpha_{r}+\frac{1}{2}\underset{m,n=i,p,s}{\sum }\frac{\partial^{2}\alpha_{r}}{\partial m\partial n}\sigma_{mn}$.

**Figure 7:**
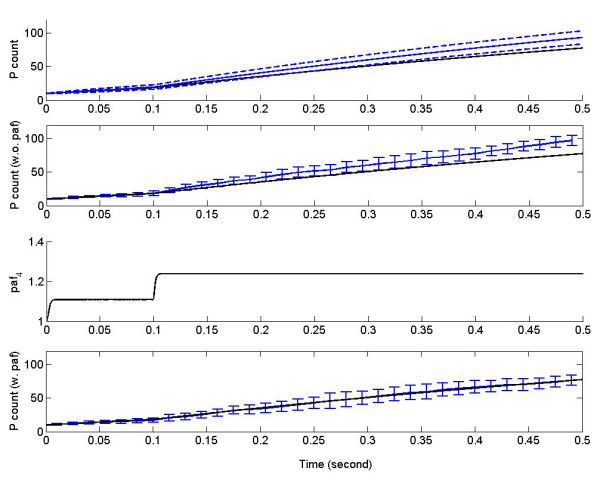
**Stochastic focusing**. The first panel from the top compares the time evolution of product molecules *P *obtained with different methods: the black line represents the solution of ODE model (64) for *P*; the blue solid line and blue dashed lines are the solutions of the moment-based model (65) for *μ_P _*and *μ_P _*± *σ_P_*, respectively. The second panel indicates that the stochastic simulations without propensity adjustment (blue error bar) diverge from the prediction by the ODE model (64) (black line). The third panel shows the propensity adjustment function *paf*4(*t*) = exp(-*f_I _f_S _*cov [log *I*(*t*), log *S*(*t*)]) for the reaction $R_{4}:I+S\overset{k_{4}}{\underset{}{\to }}S$. The bottom panel demonstrates that the propensity adjustment function *paf*_4 _achieves convergence between the stochastic simulation and the ODE model (64) (black line): the blue error bars were computed from 100 independent stochastic simulations with propensity adjustment *paf*_4_. The simulation parameters are (*i*(0), *p*(0), *s*(0), *k*_1_, *k*_2_, *k*_3_, *k*_4_, *k*_5_, *k*_6_, *f_I_*, *f_S_*) = (0, 10, 100, 10^4^, 10^3^, 1, 9.9 × 10^3^, 10^4^, 10^3^, 1.1, 0.9); at *t *= 0.1, the value of *k*_5 _changes from 10^4 ^to 0.5 ×10^4^.

The stochastic focusing model without propensity adjustment yields results quite different from those of the deterministic model, as is illustrated in Figure 7. In this figure, the blue lines in the 1^st ^panel are predicted from the moment equations (65) and the blue error bars for *μ_P _*in the 2^nd ^panel are obtained from ten independent stochastic simulations. Both diverge systematically from the black line predicted by ODE model (64). By contrast, the stochastic model with propensity adjustment produces results consistent with the deterministic model, as shown by the 4^th ^panel.

## Discussion

It is often implicitly assumed that the rate of a dynamic process can be directly taken as the propensity for a corresponding stochastic process. We have shown here that this is sometimes, but not always, true. Our results fall into three categories. The first develops conditions for a valid conversion of a rate to a propensity, the second presents a general conversion procedure, and the third discusses computational issues of propensity adjustment.

### Conditions for the direct use of a rate constant (function) as propensity function

We have shown that the direct use of a rate constant or a rate function *f *as the propensity function in a stochastic simulation algorithm requires that at least one of the following assumptions be true:

1) *f *is a linear function; this assumption has been validated in the Results sections addressing 0^th^-order and 1^st^-order reaction kinetics.

2) the reaction is monomolecular; this assumptions was evaluated in the Results section describing real-valued order monomolecular reaction kinetics.

3) all *X_i _*in the system are noise-free variables, *i.e*., without (or with ignorable) fluctuations; this assumption implies that the covariance of any two participating reactants is zero (or close to zero). This assumption is assessed in equations (29 - 36).

Each of these three conditions is a sufficient condition for the direct use of a rate function *f *as the propensity function. Moreover, these statements are valid for functions of a general format, not just for GMA. This is so because the functional formats in cases 1 and 2 above are special cases of the GMA format. For the third case, a formal proof is only given for functions in GMA format, because this structured format allows us to give an explicit estimation on how the covariance can affect the average behavior of a stochastic simulation through equation (34). For functions not in GMA format, the conclusion is still holds, although an analogous explicit estimation is lacking. The argument is as follows. The bimolecular reaction *E*[*α*_*r*_(**X**(*t*))] contains at least one quadratic moment of the form *E*[*X_i_*(*t*)*X_j_*(*t*)] (*cf*. [4] and page 38). Therefore, by definition of the covariance, *E*[*X_i_*(*t*)*X_j_*(*t*)] = *E*[*X_i_*(*t*)]*E*[*X_i_*(*t*)] + cov (*X_i_*(*t*), *X_j_*(*t*)), we obtain

$$E\left[X_{i}\left(t\right)X_{j}\left(t\right)\right]=E\left[X_{i}\left(t\right)\right]E\left[X_{j}\left(t\right)\right]\Leftrightarrow \text{cov}\left(X_{i}\left(t\right),X_{j}\left(t\right)\right)=0.$$

This result implies the following: If the covariance between every pair of random variables is zero (or ignorable), we have *E*[*X_i_*(*t*)*X_i_*(*t*)] = *E*[*X_i_*(*t*)]*E*[*X_i_*(*t*)] and therefore *E*[*α*_*r*_(**X**(*t*))] = *α*_*r*_(*E*[**X**(*t*)]). Expressed in words, the expectation of the propensity function on left-hand side of equation (29) equals its rate function, and the rate function can be directly used as propensity function in stochastic simulations.

If at least one of the three assumptions is satisfied, the stochastic simulation algorithm (SSA) is applicable without changes.

### A general procedure for converting an equation-based model into a stochastic analogue

In the past, efforts have been made to manipulate the chemical master equation (CME) in order to achieve a proper propensity function for a reduced system (*e.g*., see [2]). However, manipulations of CME are usually complicated, and successes have been modest and rare. Here we propose an alternative strategy for converting a reduced dynamical model into a stochastic analogue. To achieve this conversion, we addressed two fundamental issues: First, under what conditions can a deterministic, equation-based model be validly used in stochastic simulations? And second, what is a proper strategy to implement such a conversion?

To address the first question, we showed that the following steps are necessary:

(1) A concentration-based model needs to be converted into a particle-based model by accounting for the size of the system; if the concentration-based model is scaled (as was illustrated with the repressilator example), it may first have to be un-scaled in order to render the conversion valid;

(2) The difference between the mean of a stochastic model without propensity adjustment and the corresponding quantities of the equation-based model should be evaluated. The mean of the stochastic model is obtained either through stochastic simulations or through a moment-based approach. If the difference is significant, then an adjustment of the propensity function for a non-elementary reaction is necessary.

To answer the second question, we need to execute the following steps

(3) Compute a propensity adjustment function, either through simulated or experimental data or through a moment-based approach, in order to achieve the corrected propensity function (41);

(4) Apply SSA or one of its variants using a propensity function with adjustment to obtain valid simulation trajectories.

### Computational issues of propensity adjustments

When the propensity needs adjusting, an accurate propensity adjustment function (*paf*) is essential for obtaining the proper correction of the propensity. It is usually impossible to compute *paf *exactly, which necessitates a suitable approximation. The approximation error in *paf *originates from the following sources:

1) The expression of *paf *in Equation (40) is a function of the mean, variance, and covariance, which are computed with a 2^nd^-order Taylor expansion in log space.

2) The moment-based approach, from which the functions of mean, variance and covariance are usually derived, is an approximation method that yields a closed ODE system for the moments. In the method used here, the propensity function is approximated by a 2^nd^-order Taylor expansion, and the moments up to a certain degree (2 in our treatment) are retained, while all higher moments are assumed to be zero. One might expect that a higher-order Taylor expansion would improve the accuracy of *paf*, but it would come with a much higher computational cost. The error control of *paf *and the relative computational issues should be addressed in future studies.

Since computation cost is a major concern with the stochastic simulation of large biochemical reaction networks, another issue has yet to be addressed. Namely, how does the propensity function of a reduced system affect the accuracy and efficiency of various leaping methods that have been proposed to speed up SSA? Moreover, the question of molecular population sizes requires further analysis. Our derivation assumed large reactant populations, but simulations of a reversible pathway indicated that the method works rather well even for small populations. A more careful investigation of this issue of population size in different scenarios is still needed and should be the subject of further research.

## Conclusions

Gillespie's stochastic simulation algorithm (SSA), as well as later variants, permits three kinds of elementary reactions to be modelled: 0^th^, 1^st ^and 2^nd ^order reactions that are assumed to follow the law of mass action. All other types of reactions, containing non-integer kinetic orders and/or following other types of kinetic law, are assumed to be convertible to one of these three kinds, so that SSA can validly be applied. However, the conversion to elementary reactions is often difficult, infeasible, or simply impossible. First, the kinetic parameters of the underlying elementary reactions are in many cases unknown for a complex-order reaction. Second, even when all elementary kinetic parameters are available, the multitude of reaction channels and participating species creates a combinatorial complexity that renders SSA simulations computationally impractical. Within a deterministic framework, model reduction is a possible and often-used strategy to address such challenges. For example, a reduced mechanistic model, such as the Michaelis-Menten rate law, is often proposed to fit the experimental data, at the cost of sacrificing the original mechanistic interpretation. The reduction in these cases simplifies the original formulation by approximating, merging, or omitting intermediate reaction steps and reactants.

In this article, we propose a rather general strategy for converting a deterministic process model into a corresponding stochastic model and characterize the mathematical connections between the two. The deterministic framework is assumed to be a generalized mass action system and the stochastic analogue is in the format of the chemical master equation. The analysis identifies situations: where a direct conversion is valid; where internal noise affecting the system needs to be taken into account; and where the propensity function must be mathematically adjusted. The conversion from deterministic to stochastic models is illustrated with several representative examples, including reversible reactions with feedback controls, Michaelis-Menten enzyme kinetics, a genetic regulatory motif, and stochastic focusing. The construction of a stochastic model for a biochemical network requires the utilization of information associated with an equation-based model. The conversion strategy proposed here guides a model design process that ensures a valid transition between deterministic and stochastic models.

## Authors' contributions

JW developed the mathematical derivations, designed and performed the simulation, and drafted the manuscript. BV contributed to the statistical reasoning and revised the manuscript. EV supervised the research and revised the manuscript. All authors read and approved the final manuscript.

## Supplementary Material

Additional file 1 **Derivation of the mean and variance of a power-law function of random variables**. Click here for file

Additional file 2 **Computation of approximate mean and covariance for a generic propensity function to be used in stochastic simulations**. Click here for file
