# Supplementary material for: Constructing stochastic models from deterministic process equations by propensity adjustment
Source: BMC Syst Biol. 2011 Nov 8;5:187. doi: 10.1186/1752-0509-5-187 (PMC3236013; doi:10.1186/1752-0509-5-187)
Supplement: Additional file 1 — Derivation of the mean and variance of a power-law function of random variables. [file 1752-0509-5-187-S1.PDF]

Additional file 1

Derivation of the mean and variance of a power-law function of random variables

The computation of the expectation of a function of random variables is based on Taylor expansion. Specifically, a Taylor series is used to approximate the first two moments of a function *f* of random variable *X*, given that *f* is at least twice differentiable and the moments of *X* are finite. The result is the following:

$$\begin{aligned} & E\left[f(X)\right] \\ & = E\left[f\left(\mu_X + (X-\mu_X)\right)\right] \\ & = E\left[f(\mu_X) + f'(\mu_X)(X-\mu_X) + \frac{1}{2}f''(\mu_X)(X-\mu_X)^2 + \mathrm{HOT}(3)\right] \\ & \approx E\left[f(\mu_X) + f'(\mu_X)(X-\mu_X) + \frac{1}{2}f''(\mu_X)(X-\mu_X)^2\right] \\ & = f(\mu_X) + \frac{1}{2}f''(\mu_X)\sigma_X{}^2. \end{aligned} \tag{A.1}$$

Similarly,

$$\mathrm{var}\left[f(X)\right] \approx f'(\mu_X)^2\sigma_X{}^2. \tag{A.2}$$

Here $\mu_X = E\left[X\right]$ and $\sigma_X{}^2 = E\left[(X-\mu_X)^2\right] = \mathrm{var}\left[X\right]$.

To compute the expectation of power-law functions, consider first the special case $f(X) = \log(X)$ for $X > 0$. Here, $f'(X) = X^{-1}$ and $f''(X) = -X^{-2}$ for $X > 0$. In the present context, the random number *X* represents the amount of a given molecular species, which is positive so that $f(X) = \log(X)$ is well defined. By applying the above approximation technique to log(*X*), we have

$$E\left[\log(X)\right] \approx \log(\mu_X) - \frac{1}{2}\left(\sigma_X/\mu_X\right)^2 \tag{A.3}$$

$$\mathrm{var}\left[\log(X)\right] \approx \left(\sigma_X / \mu_X\right)^2 . \tag{A.4}$$

The generic power-law function of several random variables is given as $PL(\mathbf{X}) = k\prod_{s=1}^{N_s} X_s^{\ f_s}$. The same method as before can be used to estimate its mean μ_{PL} and variance σ_{PL} , which will emerge as functions of μ_s, $\sigma_s^{\ 2}$ and $\mathrm{cov}\left[X_i, X_j\right]$, with $s, i, j = 1, \ldots, N_s$.

Being a function of random variables, the function $PL(\mathbf{X})$ is itself a random variable. Therefore, we can apply the above approximation to $PL(\mathbf{X})$:

$$E\left[\log(PL)\right] \approx \log(\mu_{PL}) - \frac{1}{2}\left(\sigma_{PL} / \mu_{PL}\right)^2 \tag{A.5}$$

$$\mathrm{var}\left[\log(PL)\right] \approx \left(\sigma_{PL} / \mu_{PL}\right)^2 . \tag{A.6}$$

Taking the log of $PL(\mathbf{X})$ we obtain

$$\log\left(PL(\mathbf{X})\right) = \log k + \sum_{s=1}^{N_s} f_s \log X_s ,$$

which allows us to consider the mean and variance of $\log\left(PL(\mathbf{X})\right)$ in log-space in the following fashion:

$$
\begin{aligned}
&E\left[\log\left(PL(\mathbf{X})\right)\right] \\
&= E\left[\log k + \sum_{s=1}^{N_s} f_s \log X_s\right] \\
&= \log k + \sum_{s=1}^{N_s} f_s \mathrm{E}\left[\log X_s\right] \\
&\approx \log k + \sum_{s=1}^{N_s} f_s \left(\log(\mu_s) - \frac{1}{2}\mu_s{}^{-2}\sigma_s{}^2\right) \\
&= \log\left(k\prod_{s=1}^{N_s} \mu_s{}^{f_s}\right) - \frac{1}{2}\sum_{s=1}^{N_s} f_s \left(\sigma_s/\mu_s\right)^2
\end{aligned}
\tag{A.7}
$$

$$
\begin{aligned}
&\mathrm{var}\left[\log\left(PL(\mathbf{X})\right)\right] \\
&= \sum_{s=1}^{N_s} f_s \mathrm{var}\left[\log X_s\right] + 2\sum_{i<j}^{N_s} \mathrm{cov}\left[f_i \log X_i, f_j \log X_j\right] \\
&\approx \sum_{s=1}^{N_s} f_s \mu_s{}^{-2}\sigma_s{}^2 + 2\sum_{i<j}^{N_s} f_i f_j \,\mathrm{cov}\left[\log X_i, \log X_j\right].
\end{aligned}
\tag{A.8}
$$

By combining (A.5)-(A.8), we have

$$
\mu_{PL} \approx k\prod_{s=1}^{N_s} \mu_s{}^{f_s} \exp\left(\sum_{i<j}^{N_s} f_i f_j \,\mathrm{cov}\left[\log X_i, \log X_j\right]\right)
\tag{A.9}
$$

$$
\sigma_{PL}{}^2 \approx \mu_{PL}{}^2 \Omega.
\tag{A.10}
$$

Here, $\Omega = \sum_{s=1}^{N_s} f_s \mu_s{}^{-2}\sigma_s{}^2 + 2\sum_{i<j}^{N_s} f_i f_j \,\mathrm{cov}\left[\log X_i, \log X_j\right]$.

In order to see the functional relationship between $\mu_{PL}, \sigma_{PL}{}^2$ and $\mu_s, \sigma_{ij} \triangleq \mathrm{cov}\left[X_i, X_j\right]$, one needs to express $\mathrm{cov}\left[\log X_i, \log X_j\right]$ as a function of μ_s and σ_{ij}. Using one of two alternative treatments of $\mathrm{cov}\left[\log X_i, \log X_j\right]$, we obtain two results, as shown below.

1) The first alternative is the approximation of $\operatorname{cov}\left[\log X_i, \log X_j\right]$, based on Taylor linearization of $\log X$ at μ_X, which ignores higher-order terms (HOT(2)). This strategy yields

$$\log X = \log \mu_X + \mu_X{}^{-1}(X - \mu_X) + \text{HOT(2)} \approx \log\left(\mu_X / e\right) + X/\mu_X \ .$$

Thus,

$$\begin{aligned}
&\log X_i \log X_j \\
&\approx \left(\log\left(\mu_i/e\right) + X_i/\mu_i\right)\left(\log\left(\mu_j/e\right) + X_j/\mu_j\right) \\
&= \log\left(\mu_i/e\right)\log\left(\mu_j/e\right) + \log\left(\mu_i/e\right) X_j/\mu_j + \log\left(\mu_j/e\right) X_i/\mu_i + \left(X_i/\mu_i\right)\left(X_j/\mu_j\right)
\end{aligned}$$

and

$$\begin{aligned}
&E\left[\log X_i \log X_j\right] \\
&= \log\left(\mu_i/e\right)\log\left(\mu_j/e\right) + \log\left(\mu_i/e\right) + \log\left(\mu_j/e\right) + E\left[\left(X_i/\mu_i\right)\left(X_j/\mu_j\right)\right] \\
&= \log \mu_i \log \mu_j + \operatorname{cov}\left[X_i/\mu_i, X_j/\mu_j\right].
\end{aligned}$$

To compute $E\left[\log X_i\right]E\left[\log X_j\right]$, we make use of the earlier approximation

$E\left[\log X\right] \approx \log \mu_X - \frac{1}{2}\left(\sigma_X/\mu_X\right)^2$:

$$\begin{aligned}
&E\left[\log X_i\right]E\left[\log X_j\right] \\
&\approx \left(\log \mu_i - \frac{1}{2}\left(\sigma_i/\mu_i\right)^2\right)\left(\log \mu_j - \frac{1}{2}\left(\sigma_j/\mu_j\right)^2\right) \\
&= \log \mu_i \log \mu_j - \frac{1}{2}\log \mu_j \left(\sigma_i/\mu_i\right)^2 - \frac{1}{2}\log \mu_i \left(\sigma_j/\mu_j\right)^2 + \frac{1}{4}\left(\sigma_i/\mu_i\right)^2\left(\sigma_j/\mu_j\right)^2 .
\end{aligned}$$

Finally,

$$
\begin{aligned}
&\operatorname{cov}\left[\log X_i, \log X_j\right] \\
&= E\left[\log X_i \log X_j\right] - E\left[\log X_i\right] E\left[\log X_j\right] \\
&\approx \left(\log\mu_i \log\mu_j + \operatorname{cov}\left[X_i/\mu_i, X_j/\mu_j\right]\right) \\
&-\left(\log\mu_i \log\mu_j - \frac{1}{2}\log\mu_j\left(\sigma_i/\mu_i\right)^2 - \frac{1}{2}\log\mu_i\left(\sigma_j/\mu_j\right)^2 + \frac{1}{4}\left(\sigma_i/\mu_i\right)^2\left(\sigma_j/\mu_j\right)^2\right) \\
&= \operatorname{cov}\left[X_i/\mu_i, X_j/\mu_j\right] \\
&+\frac{1}{2}\log(\mu_i)\left(\sigma_j/\mu_j\right)^2 + \frac{1}{2}\log(\mu_j)\left(\sigma_i/\mu_i\right)^2 - \frac{1}{4}\left(\sigma_i/\mu_i\right)^2\left(\sigma_j/\mu_j\right)^2.
\end{aligned}
\tag{A.11}
$$

Substitution of this approximation in (A.9) and (A.10) yields

$$\mu_{PL} \approx k\prod_{s=1}^{N_s}\mu_s{}^{f_s}\exp\left(-\frac{1}{2}\sum_{s=1}^{N_s} f_s\left(\sigma_s/\mu_s\right)^2 + \frac{1}{2}\Omega\right) \tag{A.12}$$

$$\sigma_{PL}{}^2 \approx \mu_{PL}{}^2\Omega, \tag{A.13}$$

where

$$
\begin{aligned}
&\Omega \approx \sum_{s=1}^{N_s} f_s\left(\sigma_s/\mu_s\right)^2 + 2\sum_{i<j}^{N_s} f_i f_j\{\operatorname{cov}\left[X_i/\mu_i, X_j/\mu_j\right] \\
&+\frac{1}{2}\log(\mu_i)\left(\sigma_j/\mu_j\right)^2 + \frac{1}{2}\log(\mu_j)\left(\sigma_i/\mu_i\right)^2 - \frac{1}{4}\left(\sigma_i/\mu_i\right)^2\left(\sigma_j/\mu_j\right)^2\}.
\end{aligned}
$$

2) An alternative way to calculate $\mathrm{cov}\left[\log X_i, \log X_j\right]$ is to assume that $(X_1,\ldots,X_s)$ is log-normally distributed (*i.e.*, $(\log X_1,\ldots,\log X_s)$ is normally distributed). Using this assumption, Law and Kelton [1] showed that

$$\mathrm{cov}\left[\log X_i, \log X_j\right] = \log\left(1+\frac{\sigma_{ij}}{\mu_i \mu_j}\right). \tag{A.14}$$

By substituting this equation into (A.9) and (A.10), one obtains

$$\mu_{PL} \approx k\prod_{s=1}^{N_s} \mu_s^{f_s} \prod_{i<j}^{N_s}\left(1+\frac{\sigma_{ij}}{\mu_i \mu_j}\right)^{f_i f_j} \tag{A.15}$$

$$\sigma_{PL}^{2} \approx \mu_{PL}^{2}\Omega. \tag{A.16}$$

Here, $\Omega = \sum_{s=1}^{N_s} f_s\left(\frac{\sigma_s}{\mu_s}\right)^2 + 2\sum_{i<j}^{N_s} f_i f_j \log\left(1+\frac{\sigma_{ij}}{\mu_i \mu_j}\right).$

The first set of expressions for μ_{PL} and σ_{PL}^{2} (A.9 and A.10) provides an easy numerical implementation if data for the computation of $\mathrm{cov}\left[\log X_i, \log X_j\right]$ are available. The second set of expressions for μ_{PL} and σ_{PL}^{2} (A.11 and A.13) gives a clear picture of how they are related to μ_s, σ_s and σ_{ij}; however, the price for this insight is the inaccuracy introduced during the

approximation. The third set (A.15 and A.16) also provides a functional form of μ_{PL} and ${\sigma_{PL}}^2$ on $(\mu_s, \sigma_s, \sigma_{ij})$, but requires the assumption of log- normality.

Reference

1. Law, A.M. and W.D. Kelton, *Simulation Modeling and Analysis*. 3 ed. 2000, Boston: Mc.Graw Hill.
