# Supplementary material for: Constructing stochastic models from deterministic process equations by propensity adjustment
Source: BMC Syst Biol. 2011 Nov 8;5:187. doi: 10.1186/1752-0509-5-187 (PMC3236013; doi:10.1186/1752-0509-5-187)
Supplement: Additional file 2 — Computation of approximate mean and covariance for a generic propensity function to be used in stochastic simulations. [file 1752-0509-5-187-S2.PDF]

Additional file 2

Computation of approximate mean and covariance for a generic propensity function to be used in stochastic simulations

Following a procedure similar to one proposed in [1], we derive an ODE system of the mean and covariance of the number of molecules. For a chemical reaction system with N_r reactions and N_s species, the governing equation is the chemical master equation (CME; Equation (21)). With simplified notation $P(\mathbf{x},t) \triangleq P(\mathbf{x},t \mid \mathbf{x}_0,t_0)$, CME becomes

$$\frac{\partial P(\mathbf{x},t)}{\partial t} = \sum_{r=1}^{N_r} \left[\alpha_r(\mathbf{x}-\mathbf{v}_r)P(\mathbf{x}-\mathbf{v}_r,t) - \alpha_r(\mathbf{x})P(\mathbf{x},t) \right]. \tag{A.1}$$

By multiplying (A.1) with x_s and then summing over all the possible states $\mathbf{x}$, we obtain

$$\sum_{\mathbf{x}} x_s \frac{\partial P(\mathbf{x},t)}{\partial t} = \sum_{r=1}^{N_r} \sum_{\mathbf{x}} x_s \left[\alpha_r(\mathbf{x}-\mathbf{v}_r)P(\mathbf{x}-\mathbf{v}_r,t) - \alpha_r(\mathbf{x})P(\mathbf{x},t) \right]. \tag{A.2}$$

Because the sum in the first term of the right-hand side covers all **x**, we are allowed to renumber terms and to replace $\mathbf{x}-\mathbf{v}_r \rightarrow \mathbf{x}$, which yields

$$\begin{aligned}
\frac{\partial E\left[X_s\right]}{\partial t} &= \sum_{r=1}^{N_r} \sum_{\mathbf{x}} \left[(x_s + \mathbf{v}_{r,s})\alpha_r(\mathbf{x})P(\mathbf{x}_r,t) - x_s \alpha_r(\mathbf{x})P(\mathbf{x},t) \right] \\
&= \sum_{r=1}^{N_r} \sum_{\mathbf{x}} \left[v_{r,s}\alpha_r(\mathbf{x})P(\mathbf{x}_r,t) \right] \\
&= \sum_{r=1}^{N_r} v_{r,s} E\left[\alpha_r(\mathbf{X})\right],
\end{aligned} \tag{A.3}$$

where $v_{r,s}$ is the sth component of vector $\mathbf{v}_r$.

In order to obtain the second central moment, we denote $\mu_s(t) = E\left[X_s(t)\right]$, multiply eqn.(A.1) by $(x_i - \mu_i)(x_j - \mu_j)$, and sum over all possible states. The result is

$$\begin{aligned}&\sum_{\mathbf{x}}(x_i-\mu_i)(x_j-\mu_j)\frac{\partial P(\mathbf{x},t)}{\partial t}\\&=\sum_{r=1}^{N_r}\sum_{\mathbf{x}}(x_i-\mu_i)(x_j-\mu_j)\left[\alpha_r(\mathbf{x}-\mathbf{v}_r)P(\mathbf{x}-\mathbf{v}_r,t)-\alpha_r(\mathbf{x})P(\mathbf{x},t)\right].\end{aligned} \tag{A.4}$$

Again transforming the first term of the left-hand side with the replacement $\mathbf{x}-\mathbf{v}_r \to \mathbf{x}$ we obtain

$$\begin{aligned}&\frac{\partial E\left[(X_i-\mu_i)(X_j-\mu_j)\right]}{\partial t}\\&=\sum_{r=1}^{N_r}\sum_{\mathbf{x}}[(x_i+v_{r,i}-\mu_i)(x_j+v_{r,j}-\mu_j)\alpha_r(\mathbf{x})P(\mathbf{x},t)\\&-(x_i-\mu_i)(x_j-\mu_j)\alpha_r(\mathbf{x})P(\mathbf{x},t)]\\&=\sum_{r=1}^{N_r}\sum_{\mathbf{x}}[v_{r,i}(x_j-\mu_j)+v_{r,j}(x_i-\mu_i)+v_{r,i}v_{r,j}]\alpha_r(\mathbf{x})P(\mathbf{x},t)\\&=\sum_{r=1}^{N_r}\left\{v_{r,i}E\left[(X_j-\mu_j)\alpha_r(\mathbf{X})\right]+v_{r,j}E\left[(X_i-\mu_i)\alpha_r(\mathbf{X})\right]+v_{r,i}v_{r,j}E\left[\alpha_r(\mathbf{X})\right]\right\},\end{aligned} \tag{A.5}$$

where $i, j = 1,\ldots,N_s$. With these results, we can now approximate the propensity function $\alpha_r(\mathbf{x})$ using a second-order Taylor expansion at $\mathbf{X} = \boldsymbol{\mu}$, which leads to the following result:

$$\alpha_r(\mathbf{x}) \approx \alpha_r(\boldsymbol{\mu}) + \sum_{s=1}^{N_s}\frac{\partial \alpha_r(\boldsymbol{\mu})}{\partial X_s}(X_s-\mu_s) + \frac{1}{2}\sum_{m,n=1}^{N_s}\frac{\partial^2\alpha_r(\boldsymbol{\mu})}{\partial X_m \partial X_n}(X_m-\mu_m)(X_n-\mu_n). \tag{A.6}$$

The approximation becomes exact when $\alpha_r(\mathbf{x})$ is a linear or quadratic function, which is the case for elementary reactions. Furthermore, its expectation is

$$E\left[\alpha_r(\mathbf{X})\right] \approx \alpha_r(\boldsymbol{\mu}) + \frac{1}{2}\sum_{m,n=1}^{N_s} \frac{\partial^2 \alpha_r(\boldsymbol{\mu})}{\partial X_m \partial X_n} E\left[(X_m - \mu_m)(X_n - \mu_n)\right]. \tag{A.7}$$

Similarly,

$$\begin{aligned} &E\left[(X_i - \mu_i)\alpha_r(\mathbf{X})\right] \\ &\approx \sum_{s=1}^{N_s} \frac{\partial \alpha_r(\boldsymbol{\mu})}{\partial X_s} E\left[(X_i - \mu_i)(X_s - \mu_s)\right] \\ &+ \frac{1}{2}\sum_{m,n=1}^{N_s} \frac{\partial^2 \alpha_r(\boldsymbol{\mu})}{\partial X_m \partial X_n} E\left[(X_i - \mu_i)(X_m - \mu_m)(X_n - \mu_n)\right]. \end{aligned} \tag{A.8}$$

Substituting eqns. (A.5) and (A.6) into (A.1) and (A.3), we obtain

$$\begin{aligned} &\frac{\partial E\left[X_s\right]}{\partial t} \\ &\approx \sum_{r=1}^{N_r} v_{r,s} \left\{ \alpha_r(\boldsymbol{\mu}) + \frac{1}{2}\sum_{m,n=1}^{N_s} \frac{\partial^2 \alpha_r(\boldsymbol{\mu})}{\partial X_m \partial X_n} E\left[(X_m - \mu_m)(X_n - \mu_n)\right] \right\} \end{aligned} \tag{A.9}$$

and

$$\begin{aligned} &\frac{\partial E\left[(X_i - \mu_i)(X_j - \mu_j)\right]}{\partial t} \\ &= \sum_{r=1}^{N_r} \; \{ v_{r,i} \sum_{s=1}^{N_s} \frac{\partial \alpha_r(\boldsymbol{\mu})}{\partial X_s} E\left[(X_j - \mu_j)(X_s - \mu_s)\right] \\ &+ v_{r,i} \frac{1}{2}\sum_{m,n=1}^{N_s} \frac{\partial^2 \alpha_r(\boldsymbol{\mu})}{\partial X_m \partial X_n} E\left[(X_j - \mu_j)(X_m - \mu_m)(X_n - \mu_n)\right] \\ &+ v_{r,j} \sum_{s=1}^{N_s} \frac{\partial \alpha_r(\boldsymbol{\mu})}{\partial X_s} E\left[(X_i - \mu_i)(X_s - \mu_s)\right] \\ &+ v_{r,j} \frac{1}{2}\sum_{m,n=1}^{N_s} \frac{\partial^2 \alpha_r(\boldsymbol{\mu})}{\partial X_m \partial X_n} E\left[(X_i - \mu_i)(X_m - \mu_m)(X_n - \mu_n)\right] \\ &+ v_{r,i} v_{r,j} \left[\alpha_r(\boldsymbol{\mu}) + \frac{1}{2}\sum_{m,n=1}^{N_s} \frac{\partial^2 \alpha_r(\boldsymbol{\mu})}{\partial X_m \partial X_n} E\left[(X_m - \mu_m)(X_n - \mu_n)\right] \right] \}. \end{aligned} \tag{A.10}$$

Finally, we denote $\sigma_{ij} = E\left[(X_i - \mu_i)(X_j - \mu_j)\right]$ and $\sigma_{ijk} = E\left[(X_i - \mu_i)(X_j - \mu_j)(X_k - \mu_k)\right]$ and obtain the mean and second central moment as

$$\frac{\partial \mu_s}{\partial t} \approx \sum_{r=1}^{N_r} v_{r,s} \left\{ \alpha_r(\boldsymbol{\mu}) + \frac{1}{2} \sum_{m,n=1}^{N_s} \frac{\partial^2 \alpha_r(\boldsymbol{\mu})}{\partial X_m \partial X_n} \sigma_{mn} \right\}$$

$$\frac{\partial \sigma_{ij}}{\partial t} \approx \sum_{r=1}^{N_r} \{ v_{r,i} \sum_{s=1}^{N_s} \frac{\partial \alpha_r(\boldsymbol{\mu})}{\partial X_s} \sigma_{js} + v_{r,j} \sum_{s=1}^{N_s} \frac{\partial \alpha_r(\boldsymbol{\mu})}{\partial X_s} \sigma_{is} + v_{r,i} v_{r,j} \left[\alpha_r(\boldsymbol{\mu}) + \frac{1}{2} \sum_{m,n=1}^{N_s} \frac{\partial^2 \alpha_r(\boldsymbol{\mu})}{\partial X_m \partial X_n} \sigma_{mn} \right]$$
$$+ v_{r,i} \frac{1}{2} \sum_{m,n=1}^{N_s} \frac{\partial^2 \alpha_r(\boldsymbol{\mu})}{\partial X_m \partial X_n} \sigma_{jmn} + v_{r,j} \frac{1}{2} \sum_{m,n=1}^{N_s} \frac{\partial^2 \alpha_r(\boldsymbol{\mu})}{\partial X_m \partial X_n} \sigma_{imn} \}.$$

If the system is assumed to have a symmetric distribution such as multivariate normal distribution [2], then the third central moment is zero and we can obtain closed-form expressions for the mean and covariance equations, namely

$$\frac{\partial \mu_s}{\partial t} \approx \sum_{r=1}^{N_r} \mathbf{v}_{r,s} \left\{ \alpha_r(\boldsymbol{\mu}) + \frac{1}{2} \sum_{m,n=1}^{N_s} \frac{\partial^2 \alpha_r(\boldsymbol{\mu})}{\partial X_m \partial X_n} \sigma_{mn} \right\} \qquad \text{(A.11)}$$

$$\frac{\partial \sigma_{ij}}{\partial t} \approx \sum_{r=1}^{N_r} \left\{ v_{r,i} \sum_{s=1}^{N_s} \frac{\partial \alpha_r(\boldsymbol{\mu})}{\partial X_s} \sigma_{js} + v_{r,j} \sum_{s=1}^{N_s} \frac{\partial \alpha_r(\boldsymbol{\mu})}{\partial X_s} \sigma_{is} + v_{r,i} v_{r,j} \left[\alpha_r(\boldsymbol{\mu}) + \frac{1}{2} \sum_{m,n=1}^{N_s} \frac{\partial^2 \alpha_r(\boldsymbol{\mu})}{\partial X_m \partial X_n} \sigma_{mn} \right] \right\}. \qquad \text{(A.12)}$$

Reference

1. Lee, C.H., K.-H. Kim, and P. Kim, *A moment closure method for stochastic reaction networks.* The Journal of chemical physics, 2009. **130**(13): p. 134107-15.
2. Kurtz, T.G., Stoch. Proc. Appl. , 1978. **6**: p. 223.
